# Effects of Xiaoyaosan on the Hippocampal Gene Expression Profile in Rats Subjected to Chronic Immobilization Stress

**DOI:** 10.3389/fpsyt.2019.00178

**Published:** 2019-04-12

**Authors:** Xiao-Hong Li, Xue-Ming Zhou, Xiao-Juan Li, Yue-Yun Liu, Qun Liu, Xiao-Ling Guo, Li-Qiang Yang, Jia-Xu Chen

**Affiliations:** ^1^School of Traditional Chinese Medicine, Beijing University of Chinese Medicine, Beijing, China; ^2^School of Basic Medical Sciences, Guangxi University of Chinese Medicine, Nanning, China; ^3^School of Basic Medical Sciences, Heilongjiang University of Chinese Medicine, Harbin, China; ^4^Formula-pattern Research Center, School of Traditional Chinese Medicine, Jinan University, Guangzhou, China

**Keywords:** Xiaoyaosan, chronic immobilization stress, the hippocampus, gene expression profile, signal pathways, network regulating gene transcription

## Abstract

**Objective:** This study examined the effect of Xiaoyaosan and its anti-stress mechanism in rats subjected to chronic immobilization stress at the whole genome level.

**Methods:** Rat whole genome expression chips (Illumina) were used to detect differences in hippocampal gene expression in rats from the control group (CN group), model group (M group) and Xiaoyaosan group (XYS group) that were subjected to chronic immobilization stress. The Gene Ontology terms and signaling pathways that were altered in the hippocampus gene expression profile were analyzed. The network regulating the transcription of the differentially expressed genes was also established. To verify the results from the gene chips, real-time quantitative polymerase chain reaction was used to determine the expression of the GABRA1, FADD, CRHR2, and CDK6 genes in hippocampal tissues. *In situ* hybridization (ISH) and immunohistochemistry were used to determine the expression of the GABRA1 and CRHR2 genes and proteins, respectively.

**Results:** Compared with the CN group, 566 differentially expressed genes were identified in the M group. Compared with the M group, 544 differentially expressed genes were identified in the XYS group. In the M and XYS groups, multiple significantly upregulated or downregulated genes functioned in various biological processes. The cytokine receptor interaction pathway was significantly inhibited in the hippocampus of the model group. The actin cytoskeleton regulation pathway was significantly increased in the hippocampus of the XYS group. The inhibition of hippocampal cell growth was the core molecular event of network regulating the transcription of the differentially expressed genes in the model group. Promotion of the regeneration of hippocampal neurons was the core molecular event of the transcriptional regulatory network in the XYS group. The levels of the GABRA1, FADD, CRHR2 and CDK6 mRNAs, and proteins were basically consistent with the results obtained from the gene chip.

**Conclusion:** XYS may have the ability of resistance to stress, enhancement immunity and promotion nerve cell regeneration by regulating the expression of multiple genes in numerous pathways and repaired the stress-induced impairments in hippocampal structure and function by inducing cytoskeletal reorganization. These results may provide the possible target spots in the treatment of stress in rats with XYS.

## Introduction

Xiaoyaosan (XYS) which was first recorded in the *Prescriptions of the Bureau of Taiping People's Welfare Pharmacy* contains Radix Bupleuri, Rhizoma Atractylodis Macrocephalae, Radix Paeoniae Alba, Poria, Radix Angelicae Sinensis, Herba Menthae, Rhizoma Zingiberis Recens, and Radix Glycyrrhizae. The long-term studies of our research group have confirmed that XYS has reliable effectiveness in preventing and curing chronic stress. XYS increases the appetite and weight of stressed rats (1) and improves the learning and memory of stressed rats (2). XYS exerts an obvious anti-depressant effect (3). At early stage, the research group studied multiple brain regions including the central hippocampus, the hypothalamus (1), the amygdala (4), the cortex and the pituitary (5), etc., in stressed rats; in particular, the hippocampus was studies using multiple research methods. XYS increases the levels of the post-synaptic density protein 95 (PSD−95) and synaptophysin (SYP) proteins in the hippocampus (2). XYS also increases the hippocampal expression of the proopiomelanocortin (POMC) (6), corticotropin releasing factor-2 (CRF-2) (5), neurotrophic protein 3 (NT3) (7), brain derived neurotrophic factor (BDNF) (8), glutamate receptor-2 (GluR2) (9), N-methyl-D-aspartic acid (NMDA) receptors subunits NR2A and NR2B (10) mRNAs and proteins, reverses the decrease in glucocorticoid receptor levels (11) in the hippocampus and decreases the hippocampal expression of the enkephalin, prodynorphin (11), glutamate receptor-1 (GluR1) (9), and tyrosine kinase B (TrkB) mRNAs and proteins (8), among other effects.

XYS exerts a bi-directional effect on the central nervous system, participates in the integrated function of the neuro-endocrine-immune network and exerts an anti-stress effect. To date, the mechanism by which XYS inhibits stress and injury in the hippocampus has not been clearly determined. Therefore, this study used an Illumina ratref-12 full-genome expression spectrum chip containing 22,226 genes to determine the profile of differentially expressed genes in the hippocampal tissues from rats subjected to chronic immobilization stress. We systematically discuss the mechanism by which XYS induces resistance to chronic stress injury in the hippocampus from the perspective of the whole genome.

## Materials and Methods

### Animals and Grouping

Sixty-nine male Sprague-Dawley rats were purchased from the Beijing Vitalriver Laboratory Animal Research Center [animal license No. SCXK (Beijing) 2006-0009]. All rats were SPF-grade and weighed 225 ± 10 g. The rats were adaptively fed for 1 week, and then randomly divided into three groups of 23 rats each: a control group (CN group), model group (M group), and Xiaoyaosan group (XYS group). Five rats from each group were placed in one cage. The rats were raised in a common animal room with a temperature of 22 ± 2°C and a relative humidity of 50–60%. The rats in each group were provided conventional feed water *ad libitum*. In this study, all animal experiments were approved by the Institutional Animal Care and Use Committee of Beijing University of Chinese Medicine and conformed to the animal welfare guideline (BUCM-4-2014070401-3001). All efforts were made to minimize animal suffering and the number of animals needed to produce reliable data.

### Drugs, Reagents, and Instruments

The Chinese herbal compound prescription used in the experiment was Xiaoyaosan which was recorded in the “Prescriptions of the Bureau of Taiping People's Welfare Pharmacy.” Xiaoyaosan consists of the following eight herbs: Chinese thorowax root (30 g), Angelica sinensis (30 g), Radix Paeoniae Alba (30 g), Rhizoma Atractylodis (30 g), Wolfiporia extensa (30 g), Radix Glycyrrhizae (15 g), Ginger (15 g), and Mint (10 g). The preparation of herbal drugs was purchased from Beijing Tongrentang Group Co., Ltd., and dissolved in solution at a concentration of 1.67 g/ml. SYBR ExscriptTM RT-PCR kits and TRIzol, PrimeScript^TM^ RT Reagent kits were purchased from TaKaRa Company (Japan). The hybridization kit and chip tester including a hybridization oven, rat expression profile chip, a chip scanner, a chip washing system, and all other reagents were provided by Illumina Company (USA). *In situ* hybridization kits for GABRA1 and CRHR2 were provided by Boster (Wuhan). Mouse anti-GABRA1 and rabbit anti-CRHR2 antibodies were provided by Abcam.

### Method Used to Establish the Model

The rat model of stress was produced by exposing the animals to chronic immobilization stress (CIS) (12). In the model and Xiaoyaosan group, rats were bound to a special binding rack for 3 h a day for 21 days. The rats in the control group were not exposed to stress. In the Xiaoyaosan group, rats were administered Xiaoyaosan via the intragastric route daily before they were subjected to the stress procedure, and the control group and model group were administered the same volume of normal saline (1/d). The emotional behavior of rats were evaluated by open field test (OFT) and Y maze experiment (YME), as shown in Supplementary Figure 1.

### Sampling

On the morning of the second day after the 21-day stress protocol, 2% sodium pentobarbital was injected into the abdominal cavity of rats to induce deep anesthesia (40 mg/kg). The rats used for gene expression and spectroscopy analyses were decapitated rapidly, the brain was removed and the hippocampus was dissected on ice on a super-clean bench. The hippocampus was placed in liquid nitrogen, and then stored in a −80°C freeze until further use. The rats used for *in situ* hybridization and immunohistochemistry were perfusion-fixed via the left ventricular ascending aorta, and then decapitated. The brain tissues were placed in 4% paraformaldehyde at 4°C for 12 h and then embedded in paraffin for subsequent use.

### Gene Chip Detection of the Differentially Genes in the Hippocampus

Total RNA was extracted from the hippocampus using TRIzol reagent. The purity and concentration of total RNA were measured with an ultraviolet spectrophotometer. Formaldehyde-denaturing agarose gel electrophoresis was performed to assess the RNA integrity. The mRNA samples extracted from hippocampal tissues from three rats per group were mixed for the chip experiment, and three biological replicates of the samples from each group were analyzed. The differentially expressed genes between the experimental and control groups were considered significant at *P* < 0.05. If the ratio of mean fluorescence intensity of the gene in the experimental group /mean fluorescence intensity of the gene in the control group was ≦0.67 or ≧1.5, the difference in expression between two specimens was considered significant (13).

### Verification of Some of the Differentially Expressed Genes in the Hippocampus Using Real-Time qPCR

The upregulation of FADD and GABRA1 genes and the downregulation of the CDK6 and CRHR2 genes were verified using real-time qPCR. All the selected RNA specimens were same as those used in the chip experiment. The Invitrogen Company synthesized the PCR primers. A 10 μl reaction was established for each gene and included 1 μl of the cDNA templates, 1 μl upstream primer, 1 μl downstream primer, and 0.5 μl SYBR Green I. The reaction conditions were set as follows: denaturation for 2 min at 95°C and 30 cycles of 94°C for 10 s, 62°C for 10 s, and 72°C for 20 s. The plate was analyzed, and then a melting curve was performed by increasing the temperature from 55 to 95°C. The reaction was terminated, and the samples were cooled to 4°C.The 2^−ΔΔ^Ct method was used to calculate the relative expression of various genes (14). Data are presented as the means ±SD; *P* < 0.05 was considered as statistically significant.

### Detection of the Hippocampal Expression of the GABRA1 and CRHR2 mRNAs and Proteins Using *in situ* Hybridization (ISH) and Immunohistochemistry, Respectively

#### *In situ* Hybridization

Sections were dewaxed with xylene and a gradient of ethanol solutions. Then, 3% H_2_O_2_ was used to inactivate the endogenous peroxidases. The mRNA nucleic acid fragments were generated by adding a freshly prepared 3% pepsase solution in citric acid to each section in a dropwise manner. Subsequently, each section was incubated with pre-hybridization liquid without probe in a 42°C incubator for 2 h. Afterwards, each section was incubated with hybridization liquid in a 42°C incubator overnight (PBS replaced the probe hybridization solution as a negative control). Next, the sections were incubated with SABC. Then, the sections were incubated with peroxidase-conjugated biotin. For detection, sections were stained with the DAB chromogen and counterstained with haematoxylin. Finally, the sections were dehydrated with ethanol, cleared with xylene, and eventually sealed with neutral gum.

#### Immunohistochemical Staining

Paraffin sections were dewaxed with xylene and a gradient of ethanol solutions. Then, sections were treated with 3% H_2_O_2_ to inactivate the endogenous peroxidases. Subsequently, sections were incubated at a high temperature and high pressure. Afterwards, sections were incubated with a blocking solution at 37°C in a humid chamber for 20 min. Sections were sequentially incubated with the primary antibody, secondary antibody, ABC and chromogen. Next, sections were counterstained. Finally, sections were dehydrated and mounted. TBS was used in place of the primary antibody as a negative control.

#### Image Processing

We chose the similar coronal sections from each group and captured images with an Olympus BX60 microscope equipped with a Nikon D700 digital camera. Using 20X objective, images of the CA_1_, CA_3_ and DG regions were captured. Image Pro Plus 6.0 image analysis software was used to analyse the images of 10 randomly selected sections from each group. Using a method described in the literature (15), the images of the hippocampal partition were processed. Before treatment, we corrected the space and optical density in microscale (the minimum scale of 0.01 mm) images and images of blank sections captured under the same conditions. The CA_1_ region was selected in a 200 × 100 μm^2^ area along the pyramidal layer, CA_3_ and DG regions were selected in 200 × 200 μm^2^ areas. The positive area and integrated optical density (IOD) of each slice was calculated, and calculate the MOD (MOD = IOD/area). The IOD represents the relative mRNA and protein levels. Data are presented as the means ± SD. *P* < 0.05 was considered to indicate statistically significant differences.

#### Bioinformatics Analysis of the Hippocampal Profile of Differentially Expressed Genes

1. The biological processes, cellular components, and molecular functions of differentially expressed genes were classified using the Database for Annotation, Visualization and Integrated Discovery (DAVID) (16, 17) and Gene Ontology (GO) (18) database. Fisher's exact probability test was employed.
2. The signaling pathways in which the differentially expressed genes were involved were classified and analyzed using Biocarta (www.biocarta.com) and Kyoto Encyclopedia of Genes and Genomes (KEGG, www.genome.jp/kegg/pathway.html) databases. The MSigDB database provided all of the pathway information. According to the hypergeometric distribution, the pathways in which significantly differentially expressed genes were involved were determined.
3. Establishment of differentially expressed transcription regulation network.
  According to the literature (19), the strategy of comparative genomics combined with promoter region sequence transcription factor binding site (TFBS) detection technology was used to establish the network regulating the transcription of the differentially expressed genes. First, the promoter sequences were collected. The data for rat promoter sequences were downloaded from the UCSC (http://genome.ucsc.edu/) genome database. Using the accession numbers of differentially expressed genes in the model and XYS groups, the promoter sequences of these differentially expressed genes were screened to predict the TFBS of potential target genes in the regulatory network. The first 20 optimal motifs were selected in each group (differentially expressed genes) of regulatory events. An analysis of the evolutionary conservation of the motifs in calculated and predicted promoter sequences was performed to identify additional potential TF regulatory regions. The analysis of the conservation of the promoter sequences in the whole genome of eight vertebrate animals was conducted using the most recent version of the UCSC database and the phast Cons software algorithm based on a Two-State Hidden Markov Model (20). For each promoter region and binding site identified using the motif probe algorithm, the corresponding conservation of the respective motif was obtained, as the PhastCons value. Then, the predicted motifs were used to perform a position weight matrix (PWM) analysis with the TFBS binding sites in the most recent transcription factor databases TRANSFAC and JASPAR using the Motif Compare algorithm (21, 22). The PWM similarity *P*-value obtained from the comparison should not exceed 10E-4 (or 10^E−4^). Meanwhile, the corresponding TF binding sequences involved in regulating gene expression were located in the conserved sequences and mapped onto the considered motif position. The score for the corresponding conserved TF binding sequences was ≧0.8 (1 represents the most conserved sequence, whereas 0 represents the least conserved sequence). The first five optimal TFs of each group were exported after the comparison. The TFs that potentially regulated the differential expression of genes in the model and XYS groups were obtained. Finally, networks regulating the cis-transcription of the differentially expressed in the model and XYS groups were established. The core genes in the network structure were calculated using the PageRank algorithm (23). The regulatory network was visualized using Cytoscape (http://www.cytoscape.org/) (24).

## Results

### Gene Chip Detection

A total of 566 differentially expressed genes were identified in the model group compared with the control group, of which 365 were upregulated and 201 were downregulated. The results are shown in Supplementary Table 1.

A total of 544 differentially expressed genes were identified in the XYS group compared with the model group, of which 265 were upregulated and 279 were downregulated. The results are shown in Supplementary Table 2.

### Go Function Analysis

In the model and XYS groups, the significant biological processes of the differentially expressed genes in the hippocampus mainly included biological regulation, immune system process, development, reproduction, multicellular organism, response to stress, and adhesion, among others. Tables 1, 2 list the names and *P*-values for biological processes that were significantly activated or suppressed, as well as the number of differentially expressed genes.

**Table 1:** Classification of the upregulated and downregulated genes involved in the significant bioprocesses of the model group compared with the control group.

	**Term**	**Genes**	***P*-value**
Up-regulated genes in model group	Biological regulation	EFNA1, DDR2, ASGR2, MAX, TTR, CDCA7, SLC2A3, SOSTDC1, TRPV4, KCNQ1, AKT3, TAAR3, FANCC, AR, BTNL8, PTGER3, OTX2, RAB4B, FADD, VAX2, VAX1, PPARGC1A, TRDN, CD38, OLR1701, MSX1, RASGRF2, ARRB2, F5, HIPK3, ARPC5L, RIN2, RIPK3, AKAP7, ZFP212, UNG, ENPP3, LOC295015, ASB13, GCGR, SNIP, ACE, SNRK, OLR1714, PRKAA2, COL8A1, NFATC3, FZD9, ERG, TRPC6, ESRRB, TBX2, CTLA4, ACACA, RPS9, TEAD2, USF2, SHOX2, RAB31, RGS1, PPP1R2, LRP6, CHRNB4, RHBDL2, CD79B, ATP6V0A4, CHRNG, CACNA1B	2.96E-02
Down -regulated genes in model group	Immune system process	RT1-CE7, CRP, STAT5B, C5, CDK6, CCL5, DBH, TERC, RT1-A3, CCL25, OAS1I, CD34, IRF7, ERAF, DEFB1	2.25E-05
	Developmental process	WNT5A, STAT5B, C5, STRN, CCL5, GJA5, TERC, HEMGN, FOXH1, ERAF, ODF4, CHM, PCDHA13, FANCA, TBPL1, KLF5, PLD2, MAFB, CCNF, CYP26A1, CDK6, DBH, PTPN12, PRM2, NFIC	3.70E-03
	Biological regulation	WNT5A, RAB3C, IL9R, PANX1, C5, STAT5B, CRP, PRND, STRN, CCL5, TERC, SCTR, HEMGN, ADPRHL1, FOXH1, STAT4, ERAF, CHM, FANCA, CEACAM1, TBPL1, KLF5, PLD2, MAFB, ZHX2, CYP26A1, OLR1504, CDK6, DBH, RT1-A3, CRHR2, NAALAD2, P2RX1, RPS6KA2, PLK1, IRF7, REM1, KCNH7, NFIC, ZFHX2, IL22RA2	5.97E-03
	Multicellular organismal process	OLR210, WNT5A, RAB3C, C5, STAT5B, STRN, GJA5, TERC, HEMGN, FOXH1, OLR482, STAT4, ERAF, CHM, ODF4, PCDHA13, FANCA, TBPL1, KLF5, PLD2, OLR693, MAFB, OLR220, CCNF, CYP26A1, OLR1504, CDK6, DBH, PTPN12, NAALAD2, P2RX1, IRF7, PRM2, NFIC	1.18E-02

**Table 2:** Classification of the upregulated and downregulated genes involved in the significant bioprocesses of the XYS group compared with the model group.

	**Term**	**Genes**	***P*-value**
Upregulated genes in XYS group	Developmental process	WNT5A, TACR3, EVX2, GRB2, C5, STRN, HEMGN, APOA5, ERAF, SOX18, GPNMB, PCDHA13, FRS2, FIGF, FANCA, RUNX3, ANAPC2, LYN, ARHGEF7, SMAD6, MYO1E, CDK6, MALT1, DBH, PRPH2, IRS1, GNAT2, ATP7A, ALOX15, UCP3, HDAC1, SPATA18, NAB1, USH1C, TGFB1I1, COL1A1, PRM2, TCF12	2.18E-04
	Multicellular organismal process	TACR3, EVX2, STRN, HEMGN, OLR1392, APOA5, ERAF, SOX18, FRS2, FANCA, ANAPC2, OLR790, LYN, ARHGEF7, LOC367539, CDK6, IRS1, SAG, MMP11, ALOX15, OLR450, SPATA18, NAB1, USH1C, OLR194, COL1A1, TGFB1I1, WNT5A, OLR1073, GRB2, C5, RPL10L, IL23A, GPNMB, PCDHA13, OLR112, FIGF, RUNX3, OLR1609, OLR220, MYO1E, SMAD6, MALT1, DBH, PRPH2, GNAT2, ATP7A, OLR1384, HDAC1, OLR1726, PRM2, TCF12	1.03E-03
	Immune system process	LYN, MYO1E, SMAD6, RT1-M1-2, C5, CXCL9, MALT1, CDK6, CXCL11, DBH, ATP7A, IL23A, ERAF	1.33E-02
	Response to stimulus	WNT5A, TACR3, OLR1073, PANX1, GRB2, C5, RT1-M1-2, CXCL9, STRN, CXCL11, IL23A, OLR1392, SLC2A1, APOA5, OLR112, OLR1609, OLR790, LYN, OLR220, SMAD6, NEIL1, TRIM25, MALT1, DBH, PRPH2, IRS1, SAG, CYP4B1, GNAT2, PCK1, ATP7A, OLR1384, HAO1, CRHR2, UCP3, HDAC1, OLR450, OLR1726, OLR194, TGFB1I1, COL1A1	4.47E-02
Down regulated genes in XYS group	Multicellular organismal process	IBSP, PCDHA6, OLR851, ERBB4, EFNA1, EFNA2, MUTED, PGAM2, DNASE1L2, EPHB3, CDH22, GPX2, OLR488, SOSTDC1, NOS3, COL8A1, TWIST1, OTOP1, FANCC, NES, PTGER3, ESRRB, SMAD5, ACACA, DLL3, ANG1, OLR247, DPYSL3, VAX1, SLCO1A5, TNNI2, MSX1, F5, ARRB2, KCNJ8, CLDN1, OLR1288, FABP1, SEMA4D, OLR855, CTNS	3.22E-03
	Developmental process	IBSP, PCDHA6, ERBB4, EFNA1, EFNA2, MUTED, DNASE1L2, EPHB3, CDH22, SOSTDC1, NOS3, COL8A1, TWIST1, OTOP1, FANCC, NES, ESRRB, SMAD5, DLL3, ANG1, DPYSL3, VAX1, SLCO1A5, MSX1, KCNJ8, CLDN1, SEMA4D, CTNS	4.58E-03
	Biological adhesion	CDH22, PCDHA6, IBSP, WISP2, F5, PCDHB3, CLDN1, LOC498972	2.8E-02

*P < 0.05 was considered to be statistically significant, and arranged in the order of the significant difference. Genes: The differential genes which were screened in the bioprocess in this experiment*.

### Signaling Pathway in Which the Differentially Expressed Genes Were Involved

**Figure 1:**
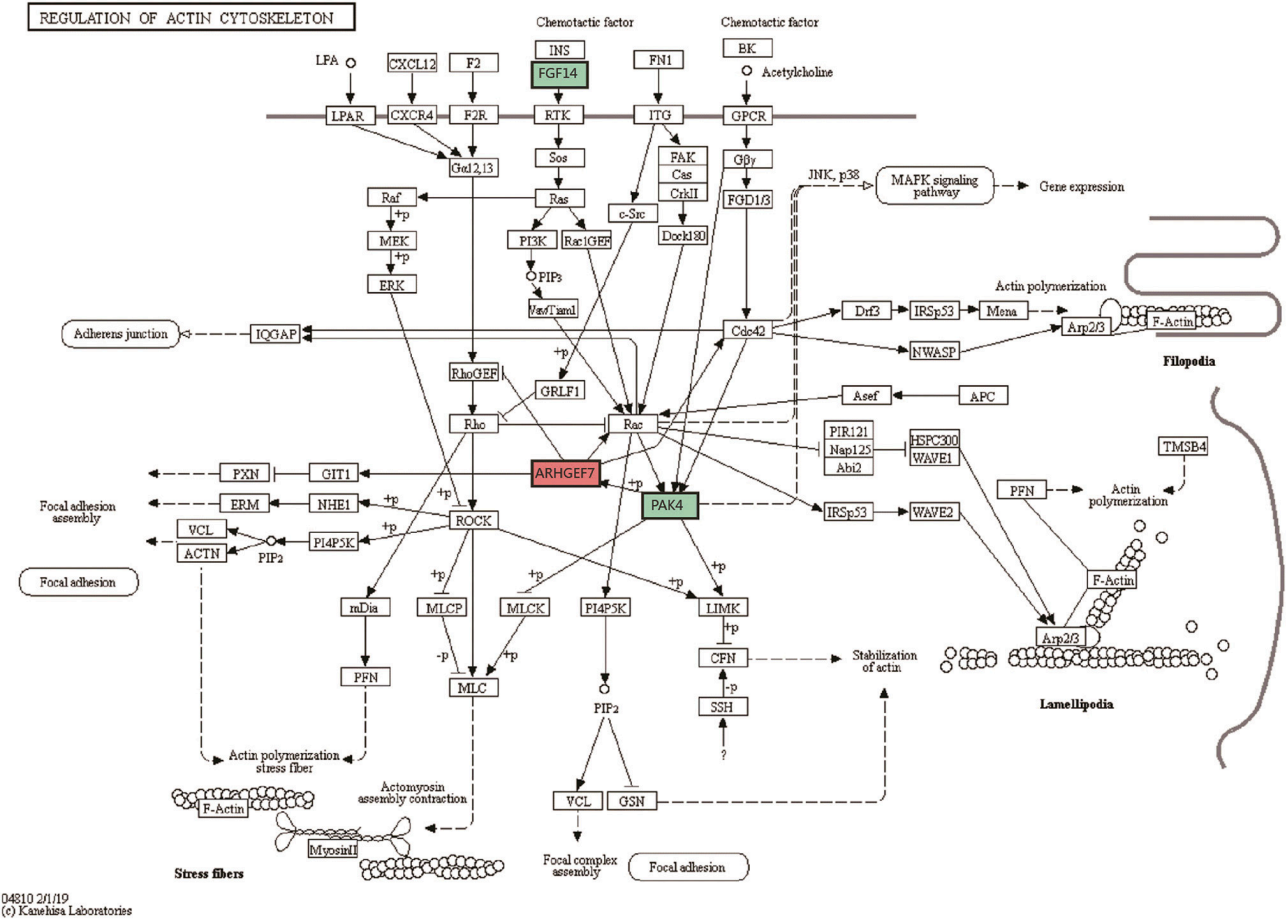
Signaling pathways regulating the actin cytoskeleton. Diagram of the most significant signaling pathways in which the differentially expressed genes in the XYS group were involved compared with the model group. The green label represents the downregulated FGF14 and PAK4 genes; the red label represents the upregulated ARHGEF7 gene.

Compared with the control group, differentially expressed genes were involved in 4 significant signaling pathways in the model group. The function of the cytokine-cytokine receptor interaction pathway was significantly inhibited, with one upregulated gene and four downregulated genes. The upregulated gene was IL17RB. The downregulated genes were IL22RA2, IL9R, CCL5, and CCL25. The pathway diagram has been published in the Li et al. (19). Compared with the model group, differentially expressed genes in the XYS group were involved in 4 significant pathways. The actin cytoskeleton regulation pathway changed significantly, with upregulation of the ARHGEF7 gene and downregulation of the FGF14 and PAK4 genes. The pathway diagram is shown in Figure 1. Tables 3, 4 list all the significant signaling pathways in which the differentially expressed genes in the model and XYS groups were involved (the pathways with the HAS prefix were obtained from the KEGG database, and the others without a unified prefix were obtained from the Biocarta database), the number of genes in each pathway in the database, the number of differentially expressed genes, the names of upregulated genes and downregulated genes and *P*-values are shown.

**Table 3:** Analysis of the differential gene pathways of model group compared with the control group.

**Pathways**	**Genes in geneset (K)**	**Genes in overlap (k)**	**Up-regulated Genes**	**Down-regulated Genes**	***P*-value**
HSA04060-CYTOKINE_CYTOKINE_RECEPTOR_INTERACTION	257	5	IL17RB	IL22RA2, IL9R, CCL5, CCL25	1.98E-02
HSA04810_REGULATION_OF_ACTIN_CYTOSKELETON	212	4	ITGB6, FGF14 ARPC5	ITGA10	2.95E-02
ACE_INHIBITOR	8	2	AGTR1, ACE		3.92E-02
SIG_PIP3_SIGNALING_IN_B_ YMPHOCYTES	36	4	ITPR2, AKT3 DAPP1	RPS6KA2	4.82E-02

**Table 4:** Analysis of the differential gene pathways of XYS group compared with the model group.

**Pathways**	**Genes in geneset (K)**	**Genes in overlap (k)**	**Up-regulated Genes**	**Down-regulated Genes**	***P*-value**
HSA04810_REGULATION_OF_ACTIN_CYTOSKELETON	212	3	ARHGEF7	FGF14 PAK4	2.71E-02
TERTPATHWAY	8	2	HDAC1	MAX	3.10E-02
CALCIUM_REGULATION_IN_CARDIAC_CELLS	143	3	FXYD2	ARRB2, RGS1	4.34E-02
HSA04060_CYTOKINE_CYTOKINE_RECEPTOR_INTERACTION	257	6	IL22RA2, CNTFR, IL23A, CXCL11, CXCL9, LTBR		4.49E-02
GPCRDB_CLASS_A_RHODOPSIN_LIKE	185	3		PTGER1, CNR1, GPR30	5.14E-02
IL4PATHWAY	11	2	GRB2, IRS1		5.43E-02

*P < 0.05 was considered to be statistically significant, and arranged in the order of the significant difference genes in geneset (K): the number of genes in this pathway in the database; genes in overlap (k): the number of differential genes screened in this experiment. Up-regulated Genes and Down-regulated Genes: The up-regulated and down-regulated genes of the differential genes on the pathway in the experiment*.

### Establishment of Networks Regulating the Transcription of the Differentially Expressed Genes

(1) Compared with the control group, 39 genes were involved in the establishment of transcriptional regulatory networks for the 566 differentially expressed genes in the model group, of which 22 were upregulated and 17 were downregulated.

**Figure 2:**
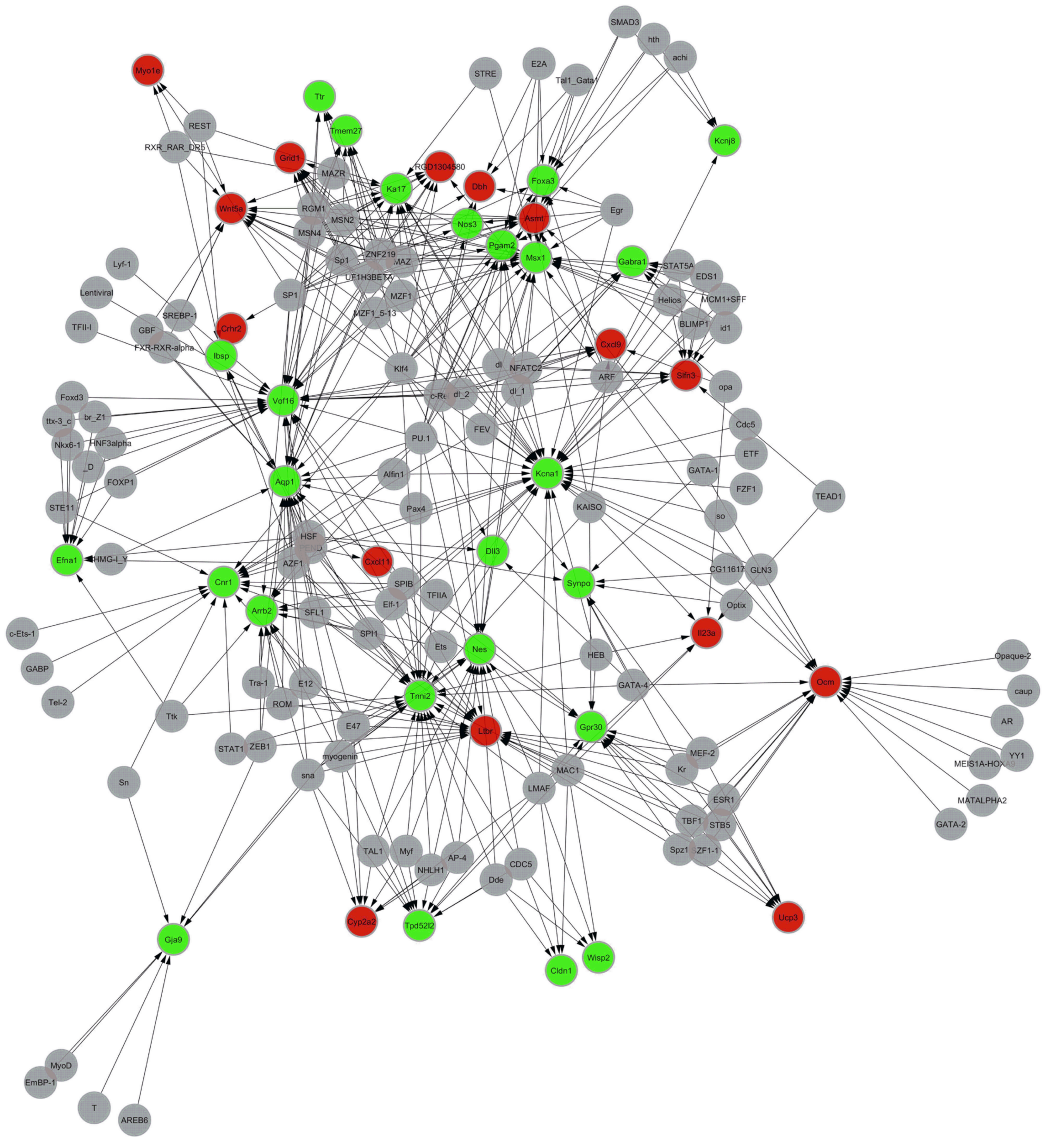
Diagram of the networks regulating the transcription of the differentially expressed genes in the model group compared with the XYS group. Red and green represent upregulated and downregulated genes, respectively, and gray represents TFs. The arrow indicates the direction in which the TF target (TFT) genes are regulated by transcription factors (TFs).

One hundred two transcription factors (TF) were predicted. One hundred forty-one nodes (including transcription factors and target genes) and 384 pairs of TF → RTFT potential relationships were identified in the transcriptional regulatory network. (2) Compared with the model group, 40 genes were involved in the establishment of the transcriptional regulatory networks for the 544 differentially expressed genes in the XYS group, of which 15 were upregulated and 25 were downregulated. One hundred twelve transcription factors (TF) were predicted. One hundred fifty-two nodes (including transcription factors and target genes) and 478 pairs of TF → RTFT potential relationships were identified in the transcriptional regulatory network. Table 5 lists the PageRank values of the first 10 core genes in the network structure and their common TFs. Figure 2 show the diagrams of the regulatory network structures. Red and green represent upregulated and downregulated genes, respectively, and gray represents TFs. The direction of regulation between the TF and TFT is presented as an arrow.

**Table 5:** PageRank values of the first 10 core genes in the network structure diagram and their common TFs.

**Gene**	**Transcription factor**	**PageRank**
**M-N**		
Klf5	AP-2,v-Maf,LMAF,SRY,odd,Myf,AP-2alphaA,HAP5, COMP1,HEN1,NFE2L2,AZF1,NHP10,CAC-binding,MAC1, bZIP910,Dde,Nrf-2,Adf-1	0.052951
Lfng	SWI5,E2A,Adf-1,myogenin,AP-2alphaA,LMAF,GAMYB, SMAD3,Myf,Spz1, AP-2, ARF, LBP-1,MET32,hth,achi, REST,odd,HEB,HEN1,AP-4,Kr, MyoD, MyoD,CAC-binding, NHP10	0.049318
Cryba2	Dfd,bZIP910,NRSF,INO2,AtMYB-15,c-Ets-1,INO4, Osf2	0.043825
Pter	Achi,Dde,Lmo2,ZEB1,sna,TBX5,E2A,Brachyury, MyoD,T,LMAF,SMAD3,CACD,MAC1,LF-A1	0.0403
Ppargc1a	TAL1,SP1,MAZ, AP-4,RAP1,Tra-1,TFII-I,SPIB,ISGF-3 CUP2,Retroviral,PEND,myogenin,PU.1,AZF1, EWSR1-FLI1,UF1H3BETA, Klf4,LBP-1,NHLH1,Elf-1,Dfd,E47,sna,SFL1, CAC-binding,Myf, Dde	0.039645
Krt1-19	Achi,TFII-I,EWSR1-FLI1,SP1,E2A,ARF,Skn-1,HTF, SMAD3,Oct-1, Opaque-2, MATALPHA2	0.036807
Tacstd2	Kr,Dde,E2A,MAC1,LMAF,HSF,TBF1,MEF-2,ESR1, SZF1-1,RAP1,Cdx,LUN-1,LF-A1	0.033357
Nxf	ARF,MEF-2,KR,PEND,E2A, Retroviral,Ttk,SPIB,SFL1, TBF1,SZF1-1,LUN-1,CUP2,Elf-1,HSF,PU.1,AZF1	0.031067
Tgm1	NHLH1,Sp1,AP-4,E47,myogenin,GC,AZF1, PEND,Klf4,sna,KROX,TAL1,SUT1, Myf,DAL81,LBP-1,Elf-1	0.025603
Cyp26a1	LMAF,bZIP910,bZIP911,c-Myc,sna,MAC1,TAL1,E47, LF-A1,NF-muE1,E12,Dde,HAP5	0.025388
**XYS-M**		
Ocm	MEF-2, Caup, STB5, GATA-1, MATALPHA2, GATA-2, GLN3, GATA-4, Kr, Spz1, MEIS1A-HOXA9, SZF1-1, CG11617, Opaque-2, YY1, ARF, ESR1, AR, Optix, TBF1	0.061146
Vof16	AZ, PEND, Elf-1, AZF1, HSF, MSN4, Lentiviral, ARF, SPIB, ttx-3_c, PU.1, Lyf-1, NFATC2 _D, ZNF219, c-Rel, MAZR, Nkx6-1, Foxd3, UF1H3BETA, SFL1, HNF3alpha, STE11, MSN2, MZF1, TFII-I, SP1, Dl, FOXP1, TFIIA, dl_1, br_Z1, MZF1_5-13, RGM1, dl_2	0.05971
Kcna1	Alfin1, Ets, Pax4, MZF1_5-13, MZF1, PU.1, SPIB, Optix, ETF, MAZ, BLIMP1, Egr, MCM1+SFF, LMAF, NFATC2, SP1, EDS1, SPI1, KAISO, Dl, Elf-1, So, PEND, AZF1, id1, STAT5A, UF1H3BETA, Helios, CG11617, dl_1, FEV, ZNF219, FZF1, E47, Cdc5, GLN3, HEB, MAC1	0.05841
Aqp1	PU.1, Sp1, AZF1, MAZ, Elf-1, FXR-RXR-alpha, Tra-1, ZEB1, PEND, ZNF219, GBF, ROM, UF1H3BETA, E47, SREBP-1, E12, SFL1, MSN4, RGM1, Myogenin, MSN2, Pax4, HMG-I_Y, Klf4, SPIB, Sna, SPI1, Alfin1	0.04028
Cnr1	CDC5, LMAF, Dde, MAC1	0.033435
Tnni2	PEND, Klf4, LMAF, Ets, MAC1, SPIB, AP-4, Myf, PU.1, TFIIA, AZF1, Myogenin, Ttk, E47, TAL1, GATA-4, KAISO, HEB, SFL1, Elf-1, SPI1, STAT1, CDC5, Sna, Dde, NHLH1, HSF	0.03207
Msx1	FEV, dl_1, Sp1, RGM1, ZNF219, NFATC2, MZF1, PU.1, STRE, MSN4, Dl, Helios, STAT5A, MSN2, E2A, id1, Klf4, EDS1, MAZ, Egr, BLIMP1, UF1H3BETA, GATA-1, MZF1_5-13, MCM1+SFF, MAZR, Tal1_Gata1	0.030168
Gja9	ZEB1, Sn, E47, AREB6, EmBP-1, T, MyoD, sna	0.030136
Efna1	FOXP1, STE11, HSF, Foxd3, Nkx6-1, ttx-3_c, Ttk, AZF1, br_Z1, HNF3alpha_D	0.027062
Ltbr	Kr, SZF1-1, SPI1, ESR1, STB5, MEF-2, SPIB, sna, E47, PU.1, Spz1, ROM, E12, myogenin, PEND, TBF1, Elf-1, SFL1, Tra-1, AZF1, ZEB1	0.02606

*The names of the first 10 core genes in the model group and XYS group in the network structure in descending order according to the PageRank value. Transcription factor column lists the predicted transcription factor of regulating and controlling each core gene*.

### Real-Time Fluorescence Quantitative PCR

**Figure 3:**
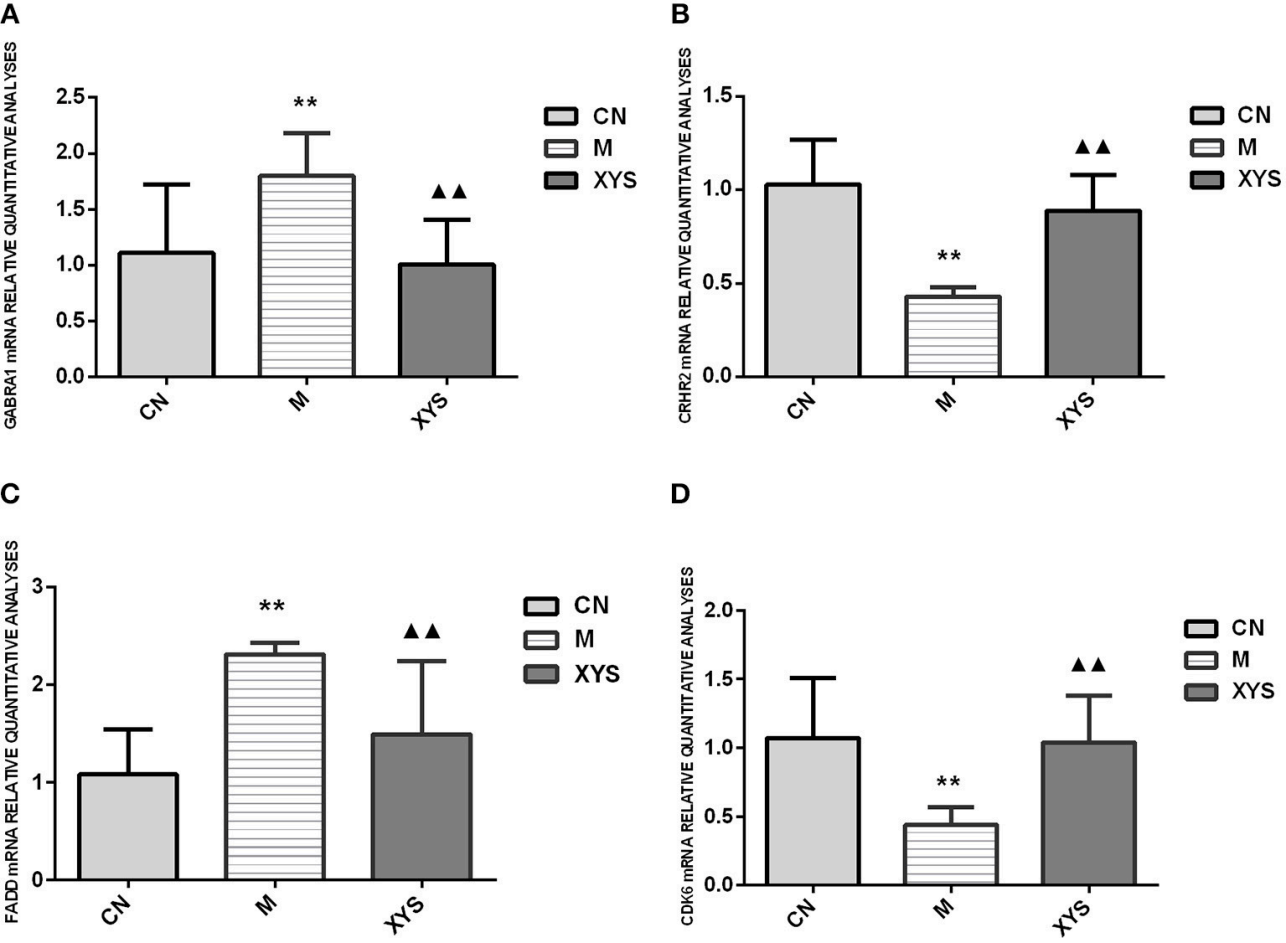
Relative quantitative analyses of the expression of the GABRA1, CRHR2, FADD, and CDK6 mRNAs in the rat hippocampal tissues from each group. **(A)** Relative quantitative analyses of GABRA1 mRNA expression in each group. **(B)** Relative quantitative analyses of CRHR2 mRNA expression in each group. **(C)** Relative quantitative analyses of FADD mRNA expression in each group. **(D)** Relative quantitative analyses of CDK6 mRNA expression in each group. ^**^P < 0.01 compared with the control group. ^▴▴^*P* < 0.01 compared with the model group.

The 2^−ΔΔ*Ct*^ method was used to compute the relative expression of the verified gene. The ratio of the mean fluorescence intensities of the genes in the gene chip experimental group to the mean fluorescence intensities of the genes in the control group served as relative parameter to confirm the data. In the relative quantitative analysis of real-time PCR data, a relative expression ratio of the experimental group to the control group of ≦1.5 or ≧0.67 suggested that the trends for the upregulation or downregulation of gene expression, respectively, were consistent with the gene chip results. The difference in the relative expression of each gene between groups was statistically analyzed, and the results were basically consistent with the differentially expressed genes identified using the gene chip (Figure 3).

### Effects of Xiaoyaosan on the Expression of the CRHR2 and GABRA1 Proteins and m RNAs in the Rat Hippocampus

**Figure 4:**
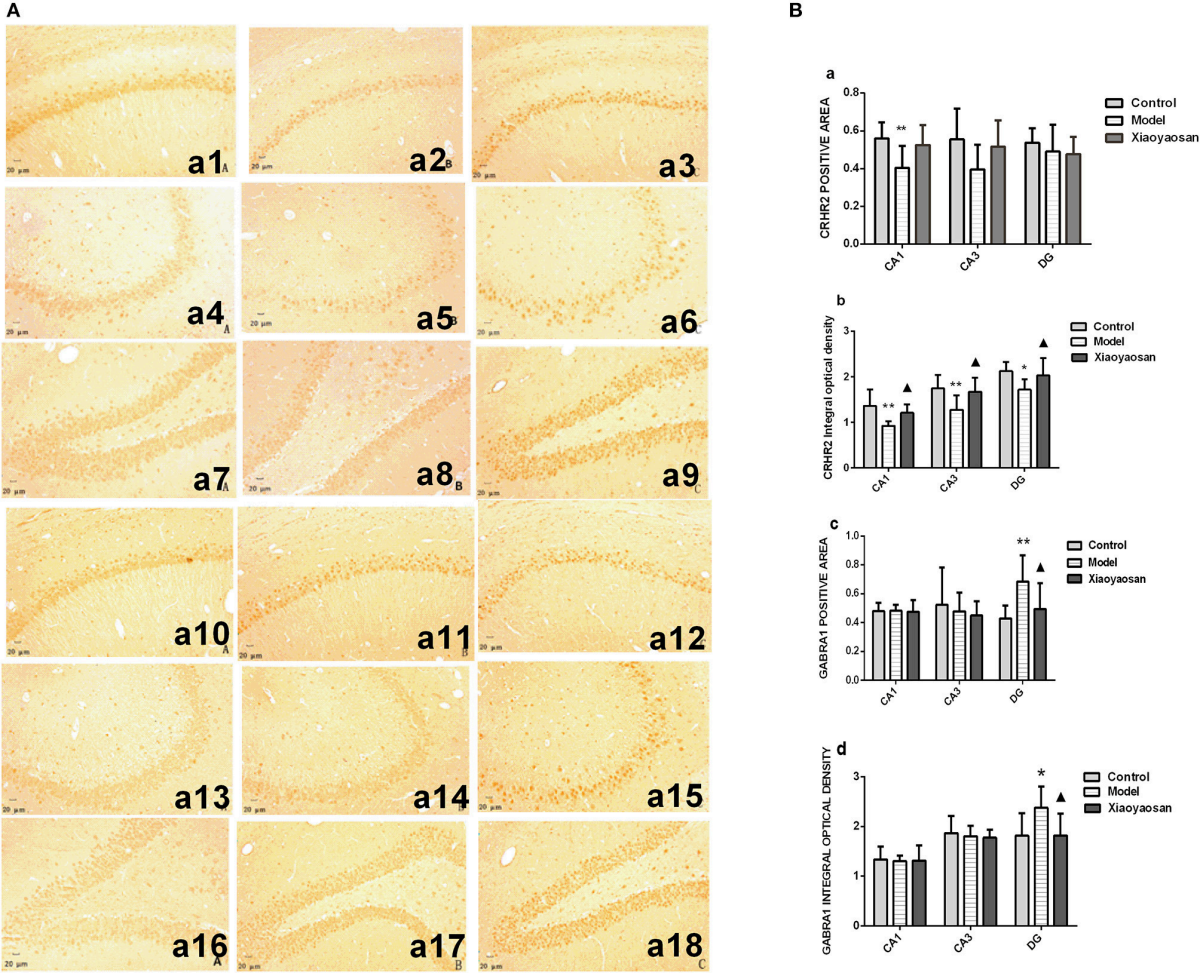
Effects of Xiaoyaosan on the expression of the CRHR2 and GABRA1 mRNAs in the rat hippocampus. **(A)** The expression of the CRHR2 and GABRA1 mRNAs in the hippocampal CA_1_, CA_3_ and DG regions. The expression of the CRHR2 mRNA in the hippocampal CA_1_ **(a1–a3)**, CA3 **(a4–a6)**, DG **(a7–a9)** regions of the control, model, and Xiaoyaosan groups is shown. The expression of the GABRA1 mRNA in the hippocampal CA_1_ **(a10–a12)**, CA3 **(a13–a15)**, DG **(a16–a18)** regions of the control, model, Xiaoyaosan groups is shown. **(B)** Changes in the expression of the CRHR2 and GABRA1 mRNAs in the rat hippocampus. **(a)** CRHR2-positive area in ISH. **(b)** Integrated optical density of the ISH for CRHR2. **(c)** GABRA1-positive area in ISH. **(d)** Integrated optical density of the GABRA1 ISH. Values are presented as the means ± SD from 10 rats per group. ^*^P < 0.05 and ^**^P < 0.01 compared with the control group. ^▴^*P* < 0.05 compared with the model group.

**Figure 5:**
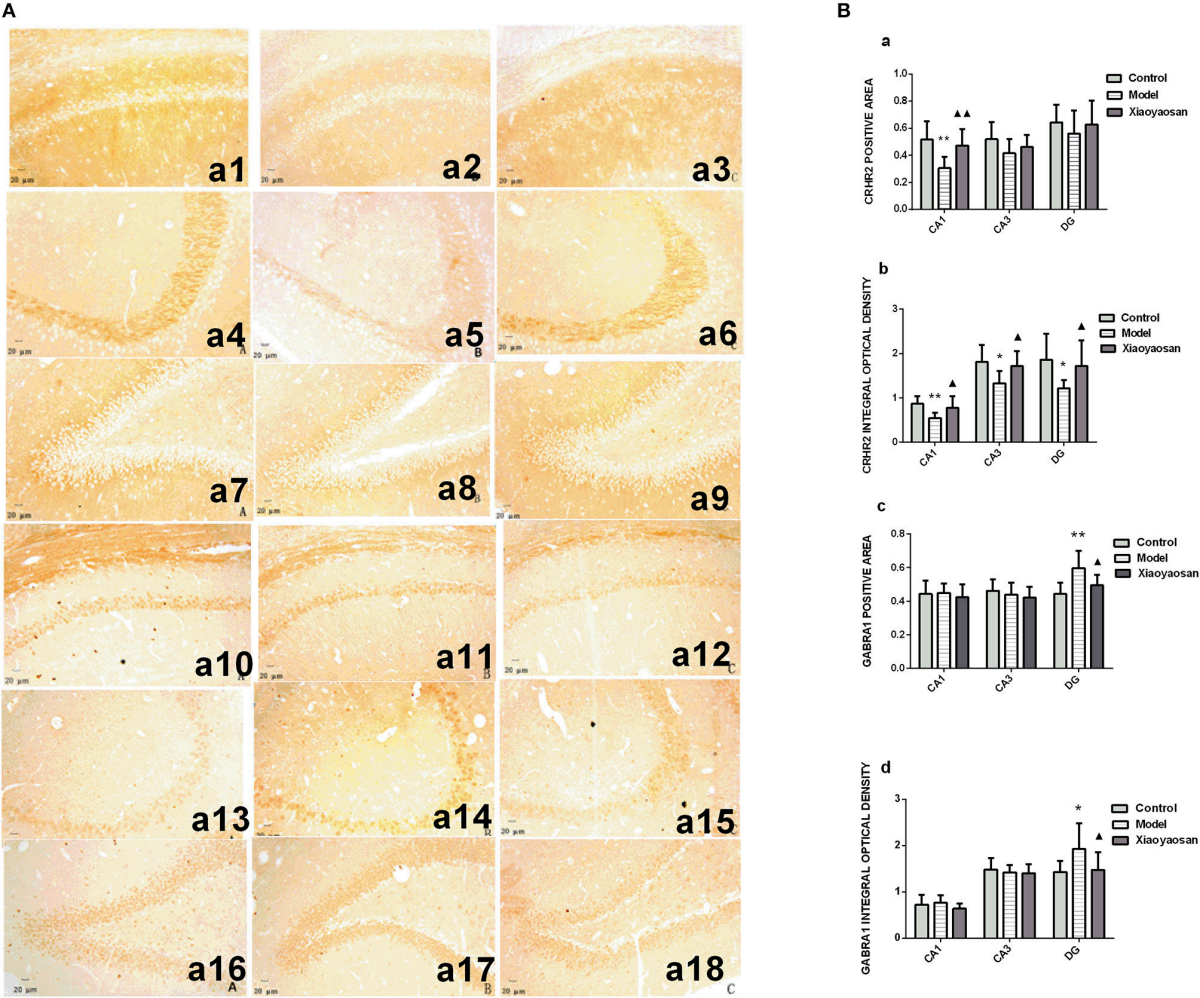
Effects of Xiaoyaosan on the levels of the CRHR2 and GABRA1 proteins in the rat hippocampus. **(A)** Levels of the CRHR2 and GABRA1 proteins in the hippocampal CA_1_, CA_3_, and DG regions. Levels of the CRHR2 protein in hippocampal CA_1_ **(a1–a3)**, CA_3_ **(a4–a6)**, DG **(a7–a9)** regions of the control, model, and Xiaoyaosan groups. Levels of the GABRA1 protein in the hippocampal CA_1_ **(a10–a12)**, CA_3_ **(a13–a15)**, DG **(a16–a18)** regions of the control, model, and Xiaoyaosan groups. **(B)** Changes in the levels of the CRHR2 and GABRA1 proteins in the rat hippocampus. **(a)** CRHR2-positive area. **(b)** Integrated optical density of the CRHR2 protein. **(c)** GABRA1-positive area. **(d)** Integrated optical density of the GABRA1 protein. Values are presented as the means±SD from 10 rats per group. ^*^P < 0.05 and ^**^P < 0.01 compared with the control group. ^▴^*P* < 0.05 and ^▴▴^*P* < 0.01 compared with the model group.

*In situ* hybridization and immunohistochemistry were used to detect the expression of the CRHR2 and GABRA1 mRNAs and proteins, respectively. The expression of the CRHR2 and GABRA1 mRNA in the hippocampal CA_1_, CA_3_ and DG regions is shown in Figure 4A. CIS reduce the area in which the CRHR2 mRNA was expressed in the hippocampal CA_1_ region (*P* < 0.01), which was reversed by the XYS treatment (*P* < 0.05) (Figure 4Ba). The integrated optical density of CRHR2 mRNA expression in the hippocampal CA_1_, CA_3_ and DG regions in model group (*P* < 0.01 or 0.05) was reduced, and this change was reversed by the XYS treatment (*P* < 0.05) (Figure 4Bb). CIS increased the area and integrated optical density of the GABRA1 mRNA in the hippocampal DG region *(P* < 0.01 or 0.05), which was reversed by the XYS treatment (*P* < 0.05; Figures 4Bc,d). Significant differences in the area and integrated optical density of GABRA1 mRNA expression in the CA_1_ and CA_3_ regions were not observed (Figures 4Bc,d). The expression of the CRHR2 and GABRA1 proteins in the hippocampal CA_1_, CA_3_ and DG regions is shown in Figure 5A.

CIS reduce the area in which the CRHR2 protein was expressed in the hippocampal CA_1_ region (*P* < 0.01), which was reversed by the XYS treatment (*P* < 0.01) (Figure 5Ba). The CRHR2-positive area showed a decreasing trend in the CA3 and DG regions, but the differences between groups were not significant (*P* > 0.05; Figure 5Ba). The integrated optical density of the CRHR2 protein was reduced in the hippocampal CA_1_, CA_3_ and DG regions (*P* < 0.01 or 0.05) of the model group, and these changes were reversed by the XYS treatment (*P* < 0.05) (Figure 5Bb). CIS increased the area and integrated optical density of GABRA1 expression in the hippocampal DG region (*P* < 0.01 or 0.05), changes that were reversed by the XYS treatment (*P* < 0.05; Figures 5Bc,d). Significant differences in the area and integrated optical density of GABRA1 expression were not observed in the CA_1_ and CA_3_ regions (Figures 5B,c,d).

## Discussion

In this study, we applied gene expression chip and bioinformatics technology to determine the central hippocampal profile of differentially expressed genes in rats exposed to CIS after the administration of the XYS intervention, and relevant research results are described above. The gene chip results for the GABRA1, FADD, CRHR2, and CDK6 genes were verified using real-time fluorescent quantitative PCR in hippocampal tissues from the rats in each group. The ISH results for the GABRA1 and CRHR2 genes, and the immunohistochemical staining for the GABRA1 and CRHR2 proteins were similar to the gene chip results. The gene chip data were verified to be reliable at the tissue and cell levels.

The most important characteristic of stress reactions is the activation of the hypothalamic-pituitary-adrenal (HPA) axis and the subsequent increase in glucocorticosteroid (GC) secretion. Activation of the HPA axis is the most important adaptative and protective response to stress, but during chronic stress, the HPA axis tends to be in a continuously highly reactive state, leading to the secretion of large amounts of GCs and the dysfunction of the nervous, endocrine, and immune systems, among others. The hippocampus is one of the most important brain regions that mediates the stress response. The hippocampus plays crucial roles in learning and memory. Because the hippocampus expresses glucocorticoid receptors (GR) at the highest levels in the central nervous system, high levels of GCs induced by stress will selectively act on the hippocampus, impairing hippocampal neuronal plasticity, disrupting the balance between apoptosis and regeneration, leading to atrophy and the loss of neurons, and eventually local damage to the hippocampal structure and function (25, 26). We genetically confirmed the reliability of previous findings. Based on the dynamic analysis of the central hippocampal profiles of differentially expressed genes in rats exposed to 21 days of chronic immobilization stress, (1) the HPA axis of the stressed rats is hyperactivated. The function of the hippocampal immune system was significantly inhibited, and the number and function of T lymphocytes were abnormal. (2) An imbalance in the synthesis and degradation of extracellular matrix (ECM) of hippocampus tissues was observed. The synthesis of ECM increased, and the degradation was reduced. Collagen synthesis was increased. The overdeposition of ECM and collagen resulted in a certain degree of hardening of the hippocampus, and the inflammatory response in the hippocampal tissue is a key factor promoting the overdeposition of ECM and collagen. (3) The balance between the growth and apoptosis of hippocampal neurons was disrupted. The growth of hippocampal neurons was inhibited, but apoptosis was accelerated. A detailed explanation of these conclusions has been published in the Li et al.

XYS is widely used as modern clinical treatment for diseases. It has been used to cure more than 165 different diseases by physicians in the fields of psychiatry, neurology, cardiology, gastroenterology, gynecology, surgery, etc., (27). It is most commonly used for psychiatric diseases and neurological diseases (28) and the most common symptoms treated are depression, followed by anxiety, cardiac symptoms, neuroses, sleep disorders, etc., (29). The regulatory effect of XYS on chronic stress has also been studied extensively. The stress-induced abnormalities in the function of the central nervous system are significantly improved or eliminated by XYS and its components, including learning and memory deficits, depression, and sleep disorders caused by psychological stress. XYS and its components also display calming, analgesic, anti-convulsant, anti-anxiety, and anti-chronic depression properties. Its effect on chronic depression is similar to imipramine (30). Research on the pharmacology of modern Chinese medicine also shows that XYS exerts a strong central pharmacological action. Among the 8 components (Radix Bupleuri, Rhizoma Atractylodis Macrocephalae, Radix Paeoniae Alba, Poria, Radix Angelicae Sinensis, Herba Menthae, Rhizoma Zingiberis Recens, and Radix Glycyrrhizae), Radix Bupleuri exhibits sedative and anti-convulsant activities, and improves central nervous excitability (31); *Angelica Sinensis* has sedative, analgesic, anti-convulsant, nerve repair, memory enhancing, anti-inflammatory, and immune boosting properties and obviously promotes the haematopoietic function of the circulatory system (32); Paeoniae has anti-inflammatory, immunomodulatory, analgesic, sedative, anti-depressant, anti-fibrotic, anti-apoptotic, and neuroprotective properties and enhances learning, and memory (33); Rhizoma Atractylodis Macrocephalae regulates the nervous and immune systems, functions as a sedative and stimulates Th1 lymphocyte growth (34); Poria exerts anti-inflammatory and sedative effects, and particularly adjusts the ratio of T cell subgroups and enhances immunity (35) Radix Glycyrrhizae enhances immune function and protects nerves (36); Rhizoma Zingiberis Recens has a dual regulatory effect on the excitation and inhibition of the central nervous system (37) Herba Menthae excites the central nervous system (38). Thus, the components of XYS regulate the function of the central nervous system.

We were encouraged to note that XYS reversed the stress-induced hippocampal damage. XYS is a multi-target, multi-pathway, and multi-channel agent with a dual regulatory function.

First, the GO analysis of the hippocampal gene expression profile in the XYS group showed that XYS restored the functions of multiple downregulated biological pathways in the stressed rats. As shown in Table 2, XYS not only regulated the downregulated developmental process and multicellular biological processes in stressed rats to ensure a new dynamic balance but also significantly inhibited the effects of and increased the function of the immune system in the stressed rats. The mechanism by which XYS regulated the immune system of the stressed rats might be related to the increased expression of the C5, DβH, ERAF, CDK6, RT1-M1-2, MALT1, SMAD6, CXCL9, CXCL11, IL23A, LYN, MYO1E, and ATP7A genes. In these upregulated genes, the expression of C5, DβH, ERAF, and CDK6 was downregulated in the model group. XYS play a direct reversal effect.

The increased expression of C5 showed that XYS reversed the complement-activated cascade reaction that was inhibited by 21 days of stress and restored the decreased humoural immune function of the stressed rats. The expression of the RT1 and M1-2 MHC class III genes was upregulated. MHC class III is mainly involved in regulating the innate immune response. Therefore, XYS not only restores the decreased humoural immunity but also improves the innate immune response of stressed rats. An increase in innate immunity is an important aspect of improving the overall immunity of the body.

Dopamine beta hydroxylase (DβH) is the key enzyme that catalzses the transformation of dopamine (DA) to norepinephrine (NE/NA). In response to stress, increased NA synthesis and the subsequent increase the capacity to adapt to the external environment mediate the adaptable regulation of the body (39, 40). XYS reversed the downregulation of DβH expression in the stressed rats and increased NA levels in the hippocampus of the stressed rats; thus, the mechanism regulating the resistance of the hippocampus to stress were enhanced.

Erythroid-associated factor (ERAF), which is also called alpha-hemoglobin-stabilizing protein (AHSP), is a protein that is expressed at high levels in erythrocytes and is closely related to the functions of these cells (41, 42). The downregulated expression of AHSP in the model group of rats revealed that 21-day CIS altered the haemopoietic system of rats. In combination with the downregulated expression of CD34 in immune system of the model group, the haemopoietic system was undoubtedly altered. The CD34 antigen is selectively expressed on the surface of haematopoietic stem cells (HSCs), progenitor cells (PCs), and endothelial cells (ECs), and promotes the formation of haematopoietic progenitor cells. XYS reversed the changes in ERAF expression during the production of erythroid cells in stressed rats, restored cell homeostasis, and maintained the function of red blood cells.

The main biological function of CDK6 is to regulate the transition between different phases of the cell cycle (43). The decreased expression of CDK6 revealed an abnormality in the cell cycle of hippocampal neurons in the stressed rats, and neuronal growth was inhibited, leading to the aging and death of neurons. XYS reversed the abnormal cell cycle of hippocampal neurons in stressed rats by up-regulating the expression of CDK6.

Here, we will focus on the expression of the SMAD6, MALT1, CXCL9, CXCL11, IL23A, LYN, MYO1E, and ATP7A genes to clarify the mechanism by which XYS restored the function of suppressed immune system. These genes were not differentially expressed in the model group, but their expression was upregulated in the XYS group.

SMAD6 is an important downstream molecule in the TGF -β/Smad signaling pathway, and it is an inhibitory Smad protein (I-Smad). The TGF-β/Smad signaling pathway has been studied extensively in a hepatic fibrosis (HF) model.ds signaling pathway has been studied extensively in hepatic fibrosis (HF) model (44). Increased Smad6 expression negatively regulates the TGFβ/Smad signaling pathway, exerts an anti-fibrotic effect (45), In addition, Smad6 blocks the SMAD signal mediated by TGF-β by blocking receptor-induced SMAD phosphorylation, thus inhibiting apoptosis (46). We observed substantial collagen deposition in the hippocampus of rats subjected to 21 days of chronic stress. The expression of some collagen proteins, such as Col8a1 (ratio: 3.17, the numbers in parentheses following each protein are all ratios), Col1a1 (1.52), Col1a1 (1.89), Col1a2 (1.53), Col3a1 (2.0), Col4a2 (1.7), and Col8a2 (1.93), was increased. Collagen inhibits cell proliferation (47, 48). The induction of collagen synthesis may promote the sclerosis of the hippocampus to some extent, resulting in the loss of some neurons. In combination with the analysis of signaling pathways, XYS not only increased the expression of the inhibitory Smad, Smad6 but also decreased the expression of the regulatory protein SMAD5 and negatively regulated the TGF-β signaling pathway (TGF_BETA_SIGNALING_PATHWAY, although the difference was not significant, *P* = 0.196). Thus, the balance between the synthesis and degradation of extracellular matrix in hippocampal neurons was restored, thus preventing hippocampal sclerosis, repairing the hippocampal damage, inhibiting the apoptosis of hippocampal neurons, increasing the number of hippocampal neurons, and restoring the suppressed immune function of hippocampus.

In addition, XYS also restored the immune function of the hippocampus by up-regulating the expression of MALT1. MALT1 is involved in the activation and function of T lymphocytes. A MALT1 deficiency reduces T cell proliferation (49), inhibits T cell activation by antigens (50). In our study, we have analyzed the number and impaired function of T lymphocytes, which is the main reason for the decrease in the immune function of stressed rats. XYS promoted the activation and proliferation of T lymphocytes by increasing the expression of MALT1.

Chemokine (C-X-C motif) ligand 9 (CXCL9) and CXCL11 are type I chemotactic factors or type Th1 chemotactic factors (51). These chemokines exert substantial effects on Th1 cell recruitment (52), and promote the production of Th1 type cytokines by decreasing the levels of Th2 cytokines. Th1 cytokines can promote the repair of normal tissues and play an important role in controlling infection and tissue damage (53, 54). In our study, the Th1/Th2 balance in the hippocampus of rats subjected to 21 days of chronic stress was disrupted, and the Th1 cells were suppressed. By increasing the expression of CXCL9 and CXCL11 (CXCL11 exhibited a significant increase, ratio: 95.67), XYS induced the accumulation of Th1 cells in the hippocampus, increased the proportion of Th1 cells in the hippocampus, and restored the Th1/Th2 cell balance. XYS not only reduced the hippocampal damage induced by Th2 cells but also promoted the repair of the hippocampal structure by increasing the number of Th1 cells. At the same time, the increased expression of CXCL9 and CXCL11 induced the accumulation of macrophages in the hippocampus by chemotaxis. As inflammatory cells, macrophages engulf and destroy the damaged tissue and help initiate the recovery process.

In addition, interleukin 23 (IL-23) and alpha subunit p19 (IL23a) increased the proportion of hippocampal Th1 cells. IL-23 is a new member of the IL-12 family that mainly functions as a proinflammatory cytokine. It promotes the proliferation of activated T cells and memory T cells and induces and activates T cells and DC to generate type Th1 cytokines, such as IFN-γ and IL-12. IL-23 causes a more persistent Th1 immune response than IL-12 (55).

In many autoimmune diseases, CXCL9 and CXCL11 are involved in the immune dysfunction in the target organs and excess amplification of the local inflammatory response in patients with various diseases (56, 57). The increased expression of CXCL9 and CXCL11 may also excessively activate the hippocampal type Th1 immune and inflammatory response and trigger autoimmune diseases, but the upregulated expression of LYN (ratio: 2.06) eliminated our concerns.

LYN is mainly expressed in inflammatory cells such as mononuclear macrophages (58). Lyn is a kinase with anti-inflammatory properties (59–61). LYN also has a very important role in maintaining the normal immune state of the body. A LYN deficiency can cause autoimmune diseases (62–64). In our study, the chronic inflammatory response in the hippocampus of the stressed rats was responsible for the decrease in the hippocampal function, and the inflammatory response was mainly a Th2 type response. Inflammation damaged the organized structure of the hippocampus, and increased the incidence of a spontaneous immune disease. An inflammatory response was also observed in the hippocampus after the administration of XYS, but it was mainly a type Th1 inflammatory response. As mentioned above, the XYS treatment activated and induced the accumulation of macrophages in the hippocampal tissue, and CXCL9 and CXCL11 induced the chemotaxis of Th1 cells to the hippocampus. The upregulated expression of LYN further may promote the activation of macrophages, enhance the inherent immune function of the hippocampus, induce the production of cytokines, and help hippocampus initiate the repair and remodeling processes. At the same time, the upregulated expression of Lyn restricted the excessive amplification of the inflammatory response. While repairing the damaged structure of the hippocampus, the Th1/Th2 cells achieved a new dynamic balance, thus avoiding the occurrence of autoimmune diseases. XYS exerted a dual regulatory effect.

MYO1E is type I myosin. MYO1E participates in numerous cell activities associated with actin fibers, such as endocytosis, signal transduction, maintenance of the cell membrane structure and tension, etc. (65), and this protein is closely related to the actin cytoskeleton. MYO1E is an indispensable component that maintains normal cellular morphology and functions (66–68). The 21-day CIS protocol increased the levels of pro-apoptosis proteins in hippocampal neurons (for example, the expression of Fadd increased) and affected the ability of hippocampal neurons to maintain a normal cytoskeleton. One of the mechanisms by which XYS restores the function of the immune system may be to increase the expression of MYO1E to repair the damaged cytoskeleton in hippocampal neurons and the damage to the structure and function of hippocampal neurons.

In addition, ATP7A is also associated with the cytoskeleton in and apoptosis of hippocampal neurons (69, 70). Increased expression of ATP7A promotes the repair of the cytoskeleton and decreases hippocampal neuron apoptosis, and it restores the structure and function of the hippocampus.

XYS reversed the core molecular events that significantly affected the structure and function of the hippocampus in stressed rats. The Th1 type inflammatory response plays an important role in the repair of the pathological damage to the hippocampal tissue in the stressed rats. The analysis of the gene regulation network and signaling pathways further confirmed the repair of the hippocampal structure and function in stressed rats.

The analysis of the gene regulation network showed that XYS reversed the pathological process of accelerated apoptosis and inhibited growth of hippocampal neurons in the stressed rats, and the regeneration of hippocampal neurons was the core molecular event in the regulatory network. The core of the network was no longer the KLF5 gene that inhibited cell growth, but the oncomodulin (OCM, Ratio was 2.03) gene that promoted cell growth. Previous studies have confirmed that confirmed that OCM was a new kind of neuronal growth factor in the central and peripheral nervous systems (71). It is an effective growth factor in the innate immune system and neurons (72). The key step in central nervous system regeneration is the growth of axons, and OCM promotes axon regeneration in the central nervous system and peripheral nervous system *in vivo* and *in vitro*. Cultured cells and the experimental mice showed that OCM obviously promotes the growth of the optic nerve, enhances the axonal regeneration capacity of the dorsal root ganglion (71, 72), and influences the direction of growth of the regenerating optic nerve axons in mice (73). Combined with the previous analysis, in our study, the increased OCM expression promoted the growth of hippocampal neurons, and OCM may also be mainly derived from activated macrophages. CXCL11 expression was significantly increased, and CXCL11 induced macrophage accumulation in the hippocampus by chemotaxis. Of course, OCM may also be derived from neutrophils, and CXCL11 can also induce granulocytes accumulation in the hippocampus by chemotaxis.

Based on the analysis of signaling pathways in hippocampal tissues from the 21-day stress group, the cytokine and cytokine receptor signaling pathway exhibited the most significant changes. We had already analyzed the upregulated IL17RB gene and downregulated IL22RA2, IL9R, CCL5, and CCL25 genes in the pathway, and the changes in the expression of these cytokines and cytokine receptors indicated that the Th2 inflammatory response predominated in the hippocampal tissues of the stressed rats. As the exposure to stress increased, the inhibitory effect on Th1 cells was more remarkable, and a lesion occurred in the hippocampal tissue (19). However, the most significant change in the XYS group occurred in the pathway regulating the actin cytoskeleton. Changes in the cytoskeleton are closely related to neuronal injury, and the extension of the axon and dendrites of neurons are related to the cytoskeleton. Significant increases in the expression of the Rho guanine nucleotide exchange factor 7 (ARHGEF7) gene, (ratio: 14.4), and significant decreases in the expression of the FGF14 (ratio: 0.43) and PAK4 (ratio: 0.56) genes in this pathway were observed.

Previous studies examining the function of RhoGEFs have frequently studied the effect on the actin cytoskeleton. ARHGEF7 regulates the actin cytoskeleton through CDC42 and PAKs (74, 75). ARHGEF7 modulates the function of PAKs that are important for regulating downstream proteins that maintain F-actin stability, such as LIM-kinases and ADF/cofilins (74, 76). ARHGEF7 affects the hyperplasia of neurites by regulating actin polymerization, and increase ARHGEF7 expression increases neurite growth (77, 78). ARHGEF7 has recently been shown to guide the actin cytoskeleton in the growth cone. The downregulation of ARHGEF7 significantly reduces neurite hyperplasia, while ARHGEF7 overexpression increases the number of neuronal growth cones (79). The increased expression of ARHGEF7 suggests that XYS induced the formation of neurites in hippocampal neurites of stressed rats and enhanced the regeneration of hippocampal neurons. By restructuring of cytoskeleton, XYS repaired the structure and function of the damaged hippocampal tissue.

P21-activated kinase 4 (PAK4) is a class II molecule in the PAKs family. PAK has many biological functions, such as regulating the cytoskeleton, cell survival and apoptosis, and the transduction and transformation of cell growth signals (80–82). Combined with the previous analysis of ARHGEF7 expression, we propose that the downregulation of PAK4 restricts the significant upregulation of ARHGEF7 expression, and the mutual interaction between the two repairs the structure and function of the hippocampal tissue.

FGF14 was downregulated in this pathway (ratio: 0.43). Previous studies on fibroblast growth factor (FGF) have focused on the central nervous system and liver fibrosis. The biological function of FGF14 is unclear. FGF14 is widely expressed in the developing and mature central nervous system, including the hippocampus, cerebral cortex (temporal lobe), putamen and cerebellum and is related to nerve signal conduction, axon transport and synaptic transmission (83). It plays important roles in spatial learning and synaptic plasticity (84, 85). We speculated that FGF14 is related to the synthesis and degradation of collagen and ECM in the hippocampus, based on the functions of FGF in the liver fibrosis model.

As mentioned above, the liver tissue of the liver fibrosis model presents obviously aberrant increase in the expression and excessive deposition of collagen fibers and extracellular matrix. FGF contributes to hepatic fibrosis. The mice that lack FGF1 and FGF2, liver fibrosis was decreased (86). Improving the expression of FGF9 in hepatic cells will lead to hepatic cell proliferation and collagen deposition (87). In patients with liver cancer, FGFs (FGF2, FGF4, FGF5, FGF9, and FGF22) are overexpressed (88). We did not identify direct reports of the role of FGF14 in hepatic fibrosis, but the FGF11-14 subfamily members interact with mitogen-activated protein kinase (MAPK) (89). The MAPK signaling pathway is closely related to the occurrence and development of fibrosis in various organs (90–92). For instance, the MAPK signaling pathway mediate the formation of liver fibrosis by regulating the activation, proliferation and apoptosis of hepatic stellate cells. Although the function of the MAPK signaling pathway was not significantly altered in response to 21 days of stress, changes in its activation or inhibition were observed. Compared with control group, the MAPK signaling pathway was activated in the 21-day stressed rats. Among the genes in the pathway, FGF14 gene (2.25), was upregulated. Compared with model group, the MAPK signaling pathway was inhibited in the 21-day XYS group. Among the genes in the pathway FGF14 (0.43), was downregulated. In this case, we hypothesized that FGF14 may be involved in the deposition and degradation of collagen and ECM in hippocampal tissue, and the downregulation of FGF14 would facilitate the degradation of collagen and the ECM in the hippocampal tissue, enabling ARHGEF to promote the growth of the hippocampal neurons and increase the number of growth cones. Of course, further studies using multiple techniques are needed to determine whether FGF14 promotes the degradation of collagen and the ECM in the hippocampal tissue under physiological and pathological conditions in the future.

In summary, the interaction of ARHGEF, Pak4, and FGF14 regulates the actin cytoskeleton in hippocampal neurons through an intricate regulatory network. ARHGEF7 positively regulates hippocampal neurons and the growth of their axons. The downregulated expression of FGF14 and Pak4 appears to inhibit the growth of hippocampal neurons, but this negative regulation balances the growth and apoptosis of hippocampal neurons. The intercoordination of positive and negative regulatory pathways enables the network of hippocampal nerve cells to be in a new equilibrium state.

In summary, we have obtained a understanding of the mechanism by which XYS enhances the resistance of the rat hippocampus to stress by bi-directionally regulating multiple genes, targets and pathways. XYS may enhance the immunocompetence and neuronal regeneration in the hippocampus of the stressed rats, and repaire the stress-induced damage to the structure and function of the hippocampus by reorganizing the cytoskeleton. The Th1 inflammatory response and CXCL11, LYN, OCM, and ARHGEF7 genes play a crucial role in the repair of the pathological damage to the hippocampus of stressed rats. XYS may exert therapeutic effects on autoimmune diseases and thalassaemia, among others. In the future, we will perform an in-depth analysis to verify the gene expression profile and provide reliable experimental evidence for the clinical application of XYS.

## Ethics Statement

In this study, all animals were carried out in accordance with the guidelines of the P. R. China legislations on the ethical use and care of laboratory animals. All efforts were made to minimize animal suffering and the number of animals needed to produce reliable data.

## Author Contributions

X-HL and X-MZ contributed equally to this work. J-XC was responsible for the conception and design of the study and the supervision of experiments and contributed to revising the manuscript. X-HL, X-MZ, X-JL, Y-YL, QL, X-LG, and L-QY performed the experiments. X-HL, X-MZ, and X-JL analyzed the data. X-MZ and X-HL contributed to the drafting of the manuscript. All authors have read and agreed with the submission of manuscript.

### Conflict of Interest Statement

The authors declare that the research was conducted in the absence of any commercial or financial relationships that could be construed as a potential conflict of interest.

## Abbreviations

AHSP: alpha-hemoglobin-stabilizing protein
ARHGEF7: Rho guanine nucleotide exchange factor 7
BDNF: brain-derived neurotrophic factor
C3: complement component 3
CIS: chronic immobilization stress
CRF-2: corticotropin releasing factor-2
Ct: cycle threshold
DA: dopamine
CXCL9: chemokine (C-X-C motif) ligand 9
DβH: dopamine beta hydroxylase
ECM: extracellular matrix
ECs: endothelial cells
ERAF: erythroid-associated factor
FGF: fibroblast growth factor
GC: glucocorticosteroid
GluR1: glutamate receptor-1
GluR2: glutamate receptor-2
GRs: glucocorticoid receptors
HF: hepatic fibrosis
HPA: hypothalamic-pituitary-adrenal
HSCs: haematopoietic stem cells
IFN: interferon
IOD: integrated optical density
IRF7: interferon regulatory factor 7
ISH: *in situ* hybridization
KLF5: Kruppel-like factor 5
LPS: lipopolysaccharide
MAPK: mitogen-activated protein kinase
MAF: macrophage activation factor
MALT1: mucosa-associated lymphoid tissue lymphoma transport protein 1
NE/NA: norepinephrine
Neg: negative
NF-κB: nuclear factor-κB
NMDA: N-methyl-D-aspartic acid
NT3: neurotrophic protein 3
OCM: oncomodulin
Pak4: p21-activated kinase 4
PCs: progenitor cells
POMC: proopiomelanocortin
Pos: positive
PSD-95: post-synaptic density protein 95
SYP: synaptophysin
TCR: T cell receptor
TF: transcription factor
TFBS: transcription factor binding site
TrkB: tyrosine kinase B
TSS: transcription start site
XYS: Xiaoyaosan.
