# Supplementary material for: Effects of Xiaoyaosan on the Hippocampal Gene Expression Profile in Rats Subjected to Chronic Immobilization Stress
Source: Front Psychiatry. 2019 Apr 12;10:178. doi: 10.3389/fpsyt.2019.00178 (PMC6474260; doi:10.3389/fpsyt.2019.00178)
Supplement: Supplementary file 1 [file Table_1.docx]

Table 1 Differentially expressed gene in the model group compared with the control group

| SYMBOL | Ratio(21M/21N) | SEARCH_KEY | TargetID |  |
| --- | --- | --- | --- | --- |
| Abca4_predicted | 1.77 | GI_62644268-S | ILMN_67394 | |
| Abo_predicted | 1.6 | GI_62644790-S | ILMN_49106 | |
| Acaca | 2.68 | GI_11559961-S | ILMN_49001 | |
| Ace | 2.15 | GI_6978756-S | ILMN_56634 | |
| Adam32 | 1.54 | GI_34879075-S | ILMN_50561 | |
| Adprhl1_predicted | 0.66 | GI_61556809-S | ILMN_59316 | |
| Agtr1 | 1.77 | GI_13591909-S | ILMN_47897 | |
| Aim1_predicted | 4.36 | GI_62666321-S | ILMN_68843 | |
| Akap10_predicted | 1.81 | GI_58865845-A | ILMN_54293 | |
| Akap7 | 1.65 | GI_62638343-A | ILMN_67729 | |
| Akr1c12_predicted | 1.6 | GI_62663781-S | ILMN_68326 | |
| Akt3 | 1.71 | GI_13928777-S | ILMN_60767 | |
| Aqp1 | 2.19 | GI_6978526-S | ILMN_53187 | |
| Ar | 1.91 | GI_6978534-S | ILMN_68782 | |
| Arid2_predicted | 1.54 | GI_62653093-S | ILMN_56766 | |
| Arpc5l_predicted | 1.57 | GI_62645046-S | ILMN_61868 | |
| Arrb2 | 1.79 | GI_6978538-S | ILMN_56088 | |
| Asb13_predicted | 1.84 | GI_62663797-S | ILMN_69366 | |
| Asgr2 | 1.51 | GI_31542149-S | ILMN_58266 | |
| Atp6v0a4_predicted | 1.66 | GI_62647030-S | ILMN_70022 | |
| Bbx_predicted | 1.61 | GI_62657842-S | ILMN_57321 | |
| Bcat1 | 1.79 | GI_8392970-S | ILMN_161861 | |
| Btnl8 | 1.89 | GI_47059098-S | ILMN_57663 | |
| C5 | 0.53 | GI_62645000-S | ILMN_59614 | |
| Ca3 | 1.66 | GI_40538863-S | ILMN_49903 | |
| Cacna1b | 1.59 | GI_25453409-A | ILMN_65757 | |
| Car12_predicted | 1.55 | GI_62653846-S | ILMN_50371 | |
| Card15_predicted | 0.6 | GI_62664934-S | ILMN_68928 | |
| Ccl25_predicted | 0.63 | GI_62658302-S | ILMN_63753 | |
| Ccl5 | 0.59 | GI_61889093-S | ILMN_53812 | |
| Ccnf | 0.4 | GI_62655817-S | ILMN_56040 | |
| Cd34_predicted | 0.6 | GI_62659990-S | ILMN_60829 | |
| Cd38 | 1.56 | GI_6978628-S | ILMN_67897 | |
| Cd79b | 2.27 | GI_57527855-S | ILMN_64333 | |
| Cdc2a | 0.43 | GI_9506474-S | ILMN_58988 | |
| Cdca7_predicted | 1.56 | GI_62645238-S | ILMN_48859 | |
| Cdh19_predicted | 1.59 | GI_57222295-A | ILMN_47944 | |
| Cdh22 | 2.24 | GI_9506476-S | ILMN_50959 | |
| Cdk6 | 0.57 | GI_62646796-S | ILMN_58924 | |
| Cdtw1 | 0.65 | GI_19705512-A | ILMN_50738 | |
| Ceacam1 | 0.31 | GI_13929059-S | ILMN_60852 | |
| Chm | 0.59 | GI_8393109-S | ILMN_62740 | |
| Chrnb4 | 2.31 | GI_16258820-S | ILMN_52337 | |
| Chrng | 1.57 | GI_9506488-S | ILMN_52509 | |
| Chsy1_predicted | 0.63 | GI_62640841-S | ILMN_56924 | |
| Cldn4_predicted | 3.47 | GI_62658763-A | ILMN_67869 | |
| Col8a1_predicted | 2.17 | GI_62657772-S | ILMN_70226 | |
| Cpn1 | 0.62 | GI_16758271-S | ILMN_62366 | |
| Cpz | 0.64 | GI_13929065-S | ILMN_57440 | |
| Crhr2 | 0.49 | GI_12083686-S | ILMN_58814 | |
| Crp | 0.63 | GI_8393196 | ILMN_51406 | |
| Cryba2 | 1.96 | GI_27465610-S | ILMN_63872 | |
| Ctla4 | 1.61 | GI_13928933-S | ILMN_54502 | |
| Ctns_predicted | 1.75 | GI_62656510-S | ILMN_53493 | |
| Cyp26a1 | 0.32 | GI_18426827-S | ILMN_51787 | |
| Dapp1_predicted | 1.66 | GI_62644457-S | ILMN_54172 | |
| Dbh | 0.56 | GI_25742779-S | ILMN_66537 | |
| Ddr2_predicted | 1.51 | GI_62659697-S | ILMN_64745 | |
| Defb1 | 0.62 | GI_13929149-S | ILMN_67018 | |
| Depdc2_predicted | 1.57 | GI_62648534-S | ILMN_51440 | |
| Dgkq_predicted | 0.66 | GI_62660014-S | ILMN_64917 | |
| Dntt_predicted | 3.57 | GI_60223046-S | ILMN_56193 | |
| Dusp18_predicted | 1.52 | GI_61557044-S | ILMN_51565 | |
| Efna1 | 1.87 | GI_16758383 | ILMN_59597 | |
| Efna2 | 1.88 | GI_62651931-S | ILMN_66783 | |
| Enpp3 | 1.68 | GI_62638345-A | ILMN_67825 | |
| Ephb3_predicted | 2.31 | GI_62658140-S | ILMN_52730 | |
| Eraf_predicted | 0.61 | GI_62641362-S | ILMN_59605 | |
| Erg | 1.91 | GI_19173755-S | ILMN_48833 | |
| Esrrb | 3.78 | GI_56606091-S | ILMN_55858 | |
| F5 | 2.3 | GI_62659616-S | ILMN_59469 | |
| Fadd | 1.96 | GI_23097353-S | ILMN_59860 | |
| Fanca_predicted | 0.21 | GI_62665410-S | ILMN_70242 | |
| Fancc | 2.12 | GI_6978828-S | ILMN_54510 | |
| Fcgr3 | 0.67 | GI_31542803-S | ILMN_54779 | |
| Fgf14 | 2.25 | GI_19224656-S | ILMN_63613 | |
| Fhl4_predicted | 1.52 | GI_61557176-S | ILMN_64188 | |
| Folr1 | 2 | GI_19424177-S | ILMN_64751 | |
| Foxh1_predicted | 0.65 | GI_62652667-S | ILMN_57947 | |
| Fscn2_predicted | 1.77 | GI_62657475-S | ILMN_67169 | |
| Fsip1_predicted | 1.52 | GI_61556875-S | ILMN_65493 | |
| Fyb_predicted | 1.71 | GI_62643003-S | ILMN_49585 | |
| Fzd9 | 1.57 | GI_23463284-S | ILMN_68013 | |
| G4 | 1.58 | GI_51571902-S | ILMN_58434 | |
| Gabra1 | 1.55 | GI_34328531-S | ILMN_53178 | |
| Gabrp | 0.67 | GI_13591944-S | ILMN_67368 | |
| Gapds | 0.57 | GI_13027413-S | ILMN_51595 | |
| Gcgr | 1.52 | GI_25742860-I | ILMN_58986 | |
| Gcl | 1.61 | GI_58219519-A | ILMN_52103 | |
| Ggt6 | 1.72 | GI_50657401-S | ILMN_51835 | |
| Gja5 | 0.53 | GI_9506724-S | ILMN_66478 | |
| Gje1 | 2.21 | GI_34871468-S | ILMN_61454 | |
| Glycam1 | 2.4 | GI_6980959-S | ILMN_53869 | |
| Golga1_predicted | 0.67 | GI_62645048-S | ILMN_61956 | |
| Golga3_predicted | 2.99 | GI_62659084-S | ILMN_65178 | |
| Gpr30 | 1.53 | GI_19424261-S | ILMN_63610 | |
| Gpr31_predicted | 0.49 | GI_27730160-S | ILMN_57736 | |
| Gzmg | 1.65 | GI_23618878-S | ILMN_57532 | |
| Hemgn | 0.65 | GI_19882214-S | ILMN_59672 | |
| Hipk3 | 2.47 | GI_13929113-S | ILMN_58063 | |
| Ifi44_predicted | 0.27 | GI_62644552-S | ILMN_58919 | |
| Ifit1 | 0.49 | GI_9910233-S | ILMN_66083 | |
| Igsf7 | 0.51 | GI_62657522-S | ILMN_48032 | |
| Iiig9 | 1.65 | GI_21955165-S | ILMN_52168 | |
| Il17rb_predicted | 1.54 | GI_62662168-S | ILMN_53866 | |
| Il22ra2 | 0.52 | GI_50979277-S | ILMN_60050 | |
| Il9r | 0.58 | GI_8393601-S | ILMN_67187 | |
| Indo | 1.6 | GI_13027383-S | ILMN_65742 | |
| Irf7_predicted | 0.65 | GI_62641611-S | ILMN_49019 | |
| Itga10_predicted | 0.52 | GI_62643928-S | ILMN_49336 | |
| Itgb6 | 1.62 | GI_51948493-S | ILMN_66282 | |
| Itpr2 | 2.14 | GI_24475710-S | ILMN_65210 | |
| Ka17 | 1.6 | GI_47087084-S | ILMN_62108 | |
| Kb9 | 0.52 | GI_57012371-S | ILMN_63509 | |
| Kcnh7 | 0.41 | GI_18777773-S | ILMN_48332 | |
| Kcnq1 | 1.65 | GI_14091761-S | ILMN_70176 | |
| Kif26a_predicted | 2.79 | GI_62651289-S | ILMN_56213 | |
| Klf5 | 0.61 | GI_16758121-S | ILMN_57735 | |
| Krt1-19 | 0.36 | GI_42409518-S | ILMN_52260 | |
| Lama1_predicted | 1.56 | GI_62655523-S | ILMN_53228 | |
| Lfng | 1.75 | GI_47575871-S | ILMN_57275 | |
| Lipogenin | 0.67 | GI_21955163-S | ILMN_61846 | |
| LOC266771 | 0.61 | GI_24308487-S | ILMN_63690 | |
| LOC287044 | 0.54 | GI_62655667-S | ILMN_65343 | |
| LOC287101 | 0.63 | GI_62655759-S | ILMN_69876 | |
| LOC287157 | 1.54 | GI_62655890-S | ILMN_60262 | |
| LOC287411 | 1.71 | GI_62656261-S | ILMN_56867 | |
| LOC287623 | 0.63 | GI_62656847-S | ILMN_48807 | |
| LOC287990 | 0.65 | GI_62658134-S | ILMN_51176 | |
| LOC288044 | 3.25 | GI_62658007-S | ILMN_66054 | |
| LOC289001 | 1.58 | GI_62659257-S | ILMN_57410 | |
| LOC290300 | 1.74 | GI_62661595-S | ILMN_51015 | |
| LOC290500 | 0.34 | GI_62662063-S | ILMN_59563 | |
| LOC290678 | 1.6 | GI_62662494-S | ILMN_54661 | |
| LOC290823 | 1.82 | GI_62662821-S | ILMN_49142 | |
| LOC290952 | 1.5 | GI_62663120-S | ILMN_64218 | |
| LOC292101 | 0.2 | GI_62665457-S | ILMN_49730 | |
| LOC292760 | 1.62 | GI_34855768-S | ILMN_55071 | |
| LOC294307 | 1.57 | GI_34852265-S | ILMN_63955 | |
| LOC294375 | 1.68 | GI_62666056-S | ILMN_55974 | |
| LOC294412 | 1.62 | GI_62666022-S | ILMN_54396 | |
| LOC294988 | 0.42 | GI_62643366-S | ILMN_67310 | |
| LOC295015 | 1.7 | GI_62644628-S | ILMN_63365 | |
| LOC295356 | 1.78 | GI_62078556-S | ILMN_62309 | |
| LOC296162 | 0.65 | GI_62645878-S | ILMN_57115 | |
| LOC296323 | 1.61 | GI_62646288-S | ILMN_54265 | |
| LOC297570 | 1.83 | GI_62648484 | ILMN_48790 | |
| LOC297890 | 1.55 | GI_62649722-S | ILMN_66870 | |
| LOC297892 | 1.87 | GI_27732752-S | ILMN_67409 | |
| LOC298014 | 1.57 | GI_62649005-S | ILMN_52369 | |
| LOC299152 | 0.66 | GI_62650896-S | ILMN_59805 | |
| LOC299458 | 0.56 | GI_27670433-S | ILMN_161737 | |
| LOC299707 | 0.35 | GI_62651989-S | ILMN_69694 | |
| LOC299820 | 1.65 | GI_27718480-S | ILMN_59025 | |
| LOC299915 | 1.8 | GI_27718848-S | ILMN_61800 | |
| LOC300128 | 0.39 | GI_62652887-S | ILMN_69082 | |
| LOC300278 | 2.79 | GI_62666694-S | ILMN_67367 | |
| LOC300742 | 1.62 | GI_62653735-S | ILMN_68051 | |
| LOC300761 | 1.53 | GI_62653775-S | ILMN_69870 | |
| LOC301388 | 1.55 | GI_62655037-S | ILMN_52383 | |
| LOC301436 | 1.81 | GI_62655090-S | ILMN_65074 | |
| LOC301518 | 0.52 | GI_27684996-S | ILMN_161763 | |
| LOC302819 | 1.59 | GI_62667379-S | ILMN_59055 | |
| LOC303373 | 1.51 | GI_27674556-S | ILMN_161745 | |
| LOC303660 | 2.15 | GI_62657305-S | ILMN_56775 | |
| LOC303887 | 0.43 | GI_62657997-S | ILMN_65442 | |
| LOC303891 | 1.5 | GI_62657978-S | ILMN_64556 | |
| LOC304037 | 1.53 | GI_62717817-S | ILMN_49839 | |
| LOC304286 | 0.65 | GI_62658452-S | ILMN_50816 | |
| LOC304530 | 2.06 | GI_62659024-S | ILMN_61455 | |
| LOC304650 | 1.87 | GI_62665036-S | ILMN_51022 | |
| LOC304918 | 1.63 | GI_62659582-S | ILMN_57786 | |
| LOC304965 | 0.42 | GI_62659713-S | ILMN_65526 | |
| LOC305078 | 1.64 | GI_62659986-S | ILMN_60637 | |
| LOC305448 | 0.55 | GI_62660640-S | ILMN_65091 | |
| LOC305898 | 1.67 | GI_62661494-S | ILMN_68402 | |
| LOC306170 | 0.5 | GI_62662045-S | ILMN_58782 | |
| LOC306577 | 2.08 | GI_62662876-S | ILMN_52655 | |
| LOC306889 | 1.99 | GI_62663532-S | ILMN_60558 | |
| LOC306891 | 1.64 | GI_27685292-S | ILMN_57067 | |
| LOC307248 | 1.64 | GI_62664656-S | ILMN_53168 | |
| LOC307660 | 1.71 | GI_62664840-A | ILMN_64310 | |
| LOC308337 | 1.65 | GI_62638862-S | ILMN_47803 | |
| LOC308990 | 1.75 | GI_62641303-S | ILMN_56832 | |
| LOC309003 | 1.6 | GI_62641344-S | ILMN_58822 | |
| LOC309100 | 1.63 | GI_62641582-S | ILMN_70269 | |
| LOC309173 | 0.59 | GI_62641768-S | ILMN_57370 | |
| LOC310212 | 0.66 | GI_34855068-S | ILMN_58655 | |
| LOC310787 | 1.76 | GI_62644198-S | ILMN_64168 | |
| LOC311404 | 0.57 | GI_62645815-S | ILMN_53995 | |
| LOC311573 | 1.79 | GI_62646205-S | ILMN_50433 | |
| LOC311591 | 1.61 | GI_62646286 | ILMN_54176 | |
| LOC311598 | 0.65 | GI_62646258-S | ILMN_52960 | |
| LOC312677 | 1.59 | GI_62648099-S | ILMN_52197 | |
| LOC312678 | 1.52 | GI_62648101-S | ILMN_52286 | |
| LOC313069 | 1.58 | GI_62648674-S | ILMN_58396 | |
| LOC313609 | 1.56 | GI_62649824-S | ILMN_49439 | |
| LOC313720 | 1.91 | GI_62650050-S | ILMN_60985 | |
| LOC313778 | 0.32 | GI_62650199-S | ILMN_68985 | |
| LOC313906 | 2.3 | GI_62650390-S | ILMN_57217 | |
| LOC314119 | 0.65 | GI_62666934-S | ILMN_56894 | |
| LOC314443 | 2.07 | GI_62651239-S | ILMN_53842 | |
| LOC314858 | 3.29 | GI_62652179-S | ILMN_56215 | |
| LOC315135 | 3.15 | GI_62652740-S | ILMN_61706 | |
| LOC315203 | 0.31 | GI_62652889-S | ILMN_69173 | |
| LOC315417 | 1.68 | GI_62653226-S | ILMN_65163 | |
| LOC315465 | 0.55 | GI_34860442-S | ILMN_47930 | |
| LOC315728 | 2.38 | GI_62653981-S | ILMN_57136 | |
| LOC316001 | 0.67 | GI_62654281-S | ILMN_49283 | |
| LOC316122 | 2.11 | GI_47058977-S | ILMN_51978 | |
| LOC316482 | 1.62 | GI_62648492-S | ILMN_49193 | |
| LOC317227 | 1.51 | GI_62667102-S | ILMN_66644 | |
| LOC317420 | 1.6 | GI_62666738-S | ILMN_69635 | |
| LOC360425 | 2.56 | GI_62652542-S | ILMN_51356 | |
| LOC360478 | 1.84 | GI_62655696-S | ILMN_66793 | |
| LOC360527 | 1.53 | GI_62656081-S | ILMN_70223 | |
| LOC360589 | 2.58 | GI_62656720-S | ILMN_64988 | |
| LOC360661 | 1.55 | GI_62657353-S | ILMN_59384 | |
| LOC360662 | 1.62 | GI_62657361-S | ILMN_59820 | |
| LOC360681 | 1.53 | GI_62657500-S | ILMN_68559 | |
| LOC360795 | 1.73 | GI_62658768-S | ILMN_68147 | |
| LOC360967 | 0.55 | GI_62660708-S | ILMN_50921 | |
| LOC361034 | 1.72 | GI_62661338-S | ILMN_53158 | |
| LOC361277 | 2.67 | GI_62663909-S | ILMN_60745 | |
| LOC361349 | 0.65 | GI_62664635-S | ILMN_52056 | |
| LOC361466 | 1.54 | GI_62638423-S | ILMN_48847 | |
| LOC361548 | 1.61 | GI_62639376-S | ILMN_52176 | |
| LOC361582 | 1.98 | GI_62639820-S | ILMN_66485 | |
| LOC361641 | 0.67 | GI_62641237-S | ILMN_53280 | |
| LOC361780 | 2.62 | GI_62642612-S | ILMN_51711 | |
| LOC361865 | 1.65 | GI_62666327-S | ILMN_69111 | |
| LOC361911 | 1.96 | GI_62643127-S | ILMN_55439 | |
| LOC361968 | 0.67 | GI_62643594-S | ILMN_55174 | |
| LOC362136 | 0.59 | GI_34854694-S | ILMN_66113 | |
| LOC362204 | 2.07 | GI_62645678-S | ILMN_47729 | |
| LOC362236 | 2.33 | GI_62646044 | ILMN_65586 | |
| LOC362437 | 0.66 | GI_62648164-S | ILMN_55453 | |
| LOC362577 | 1.62 | GI_62649538 | ILMN_57032 | |
| LOC362659 | 1.61 | GI_62650024-S | ILMN_59803 | |
| LOC362701 | 3.27 | GI_62650359-S | ILMN_55546 | |
| LOC362801 | 0.56 | GI_62651580-S | ILMN_48630 | |
| LOC362927 | 0.62 | GI_62657544-S | ILMN_55298 | |
| LOC363073 | 0.53 | GI_62653785-S | ILMN_70296 | |
| LOC363274 | 0.47 | GI_62655361-S | ILMN_65436 | |
| LOC363369 | 0.66 | GI_62718316-S | ILMN_53563 | |
| LOC363408 | 0.42 | GI_62718656-S | ILMN_51921 | |
| LOC363507 | 1.65 | GI_62667435-S | ILMN_62533 | |
| LOC363611 | 1.63 | GI_62656140-S | ILMN_50378 | |
| LOC363701 | 1.58 | GI_62657410-S | ILMN_62340 | |
| LOC363865 | 0.18 | GI_62658398-S | ILMN_70399 | |
| LOC364357 | 0.26 | GI_27699630-S | ILMN_65968 | |
| LOC364539 | 1.76 | GI_62662496-S | ILMN_54752 | |
| LOC364653 | 2.96 | GI_62663044-S | ILMN_69537 | |
| LOC364810 | 1.52 | GI_34877919-S | ILMN_161851 | |
| LOC364885 | 2.26 | GI_62664582-S | ILMN_65724 | |
| LOC365202 | 0.38 | GI_62639047-S | ILMN_57460 | |
| LOC365242 | 1.52 | GI_62639749-S | ILMN_64251 | |
| LOC365399 | 1.53 | GI_62641823 | ILMN_60324 | |
| LOC365672 | 0.57 | GI_34854235-S | ILMN_48695 | |
| LOC365982 | 1.82 | GI_34861640-S | ILMN_65408 | |
| LOC366004 | 0.48 | GI_62644878-S | ILMN_53463 | |
| LOC366078 | 0.58 | GI_62645271-S | ILMN_50518 | |
| LOC366158 | 0.64 | GI_62645552-S | ILMN_64522 | |
| LOC366254 | 1.9 | GI_62646426-S | ILMN_61691 | |
| LOC366307 | 1.51 | GI_62648602-S | ILMN_54535 | |
| LOC366550 | 1.97 | GI_62650324-S | ILMN_53928 | |
| LOC366680 | 1.73 | GI_62650931 | ILMN_61703 | |
| LOC366859 | 0.65 | GI_62651901-S | ILMN_65333 | |
| LOC367006 | 1.6 | GI_62653182-S | ILMN_62594 | |
| LOC367102 | 2.48 | GI_62653993-S | ILMN_57682 | |
| LOC367112 | 1.54 | GI_62653915-S | ILMN_53760 | |
| LOC367205 | 2.08 | GI_62654848-S | ILMN_55203 | |
| LOC367324 | 1.54 | GI_62655482-S | ILMN_49616 | |
| LOC367332 | 1.66 | GI_34877985-S | ILMN_51172 | |
| LOC367830 | 2.41 | GI_62666918-S | ILMN_56072 | |
| LOC367994 | 0.58 | GI_62658718-S | ILMN_65176 | |
| LOC368057 | 0.55 | GI_62647270-S | ILMN_58926 | |
| LOC497665 | 0.47 | GI_62655783-S | ILMN_54292 | |
| LOC497687 | 2.04 | GI_62659036-S | ILMN_62608 | |
| LOC497694 | 0.65 | GI_62660095-S | ILMN_70142 | |
| LOC497746 | 1.69 | GI_62665906-S | ILMN_49071 | |
| LOC497747 | 1.65 | GI_62642682-S | ILMN_56006 | |
| LOC497757 | 1.86 | GI_62643665-S | ILMN_58917 | |
| LOC497820 | 1.85 | GI_62652188-S | ILMN_56764 | |
| LOC497830 | 0.58 | GI_62653430-S | ILMN_52720 | |
| LOC497904 | 1.67 | GI_62656077-S | ILMN_70043 | |
| LOC498055 | 1.61 | GI_62657602-S | ILMN_58500 | |
| LOC498077 | 2.27 | GI_62657860-S | ILMN_58141 | |
| LOC498159 | 0.56 | GI_62658566-S | ILMN_57408 | |
| LOC498177 | 0.57 | GI_62658831-S | ILMN_50461 | |
| LOC498213 | 0.61 | GI_62659166-S | ILMN_49792 | |
| LOC498239 | 0.66 | GI_62659417-S | ILMN_69710 | |
| LOC498244 | 0.65 | GI_62659434-S | ILMN_47849 | |
| LOC498258 | 2.84 | GI_62659560-S | ILMN_56595 | |
| LOC498268 | 1.54 | GI_62659652-S | ILMN_61277 | |
| LOC498292 | 2.09 | GI_62659790-S | ILMN_69358 | |
| LOC498356 | 0.48 | GI_62660433-S | ILMN_70403 | |
| LOC498366 | 1.61 | GI_62660474-S | ILMN_49955 | |
| LOC498394 | 1.51 | GI_62660644-S | ILMN_65265 | |
| LOC498397 | 1.69 | GI_62660666-S | ILMN_66351 | |
| LOC498409 | 0.59 | GI_62660783-S | ILMN_55124 | |
| LOC498435 | 1.57 | GI_62660974-S | ILMN_68232 | |
| LOC498490 | 0.65 | GI_62661298-S | ILMN_51280 | |
| LOC498510 | 0.62 | GI_62661425-S | ILMN_64123 | |
| LOC498585 | 0.61 | GI_62662239-S | ILMN_57516 | |
| LOC498587 | 1.72 | GI_62662250-S | ILMN_58244 | |
| LOC498717 | 0.62 | GI_62663316-S | ILMN_58612 | |
| LOC498757 | 1.56 | GI_62663624-S | ILMN_59746 | |
| LOC498765 | 0.26 | GI_62663673-S | ILMN_62260 | |
| LOC498772 | 1.55 | GI_62663710-S | ILMN_64401 | |
| LOC498797 | 0.56 | GI_62663917-S | ILMN_61110 | |
| LOC498937 | 1.81 | GI_62665124-S | ILMN_55801 | |
| LOC498972 | 1.64 | GI_62665513-S | ILMN_52574 | |
| LOC499058 | 0.63 | GI_62638794-S | ILMN_67128 | |
| LOC499092 | 1.75 | GI_62639124-S | ILMN_61857 | |
| LOC499186 | 0.46 | GI_62640738-S | ILMN_51998 | |
| LOC499209 | 0.66 | GI_62640925-S | ILMN_61234 | |
| LOC499300 | 0.65 | GI_62641704-S | ILMN_53818 | |
| LOC499322 | 1.67 | GI_62641910-S | ILMN_64699 | |
| LOC499458 | 0.67 | GI_62666133-S | ILMN_59842 | |
| LOC499503 | 1.54 | GI_62642709-S | ILMN_57372 | |
| LOC499568 | 1.75 | GI_62643155-S | ILMN_56742 | |
| LOC499602 | 1.91 | GI_62643384-S | ILMN_68108 | |
| LOC499758 | 1.59 | GI_62644767-S | ILMN_48001 | |
| LOC499781 | 1.92 | GI_62644952-S | ILMN_57290 | |
| LOC499818 | 2.01 | GI_62645325-S | ILMN_53289 | |
| LOC499865 | 1.85 | GI_62645568-S | ILMN_65229 | |
| LOC499893 | 0.43 | GI_62645898-S | ILMN_58207 | |
| LOC499957 | 0.2 | GI_62646554-S | ILMN_68529 | |
| LOC499985 | 0.6 | GI_62646700-S | ILMN_54350 | |
| LOC500010 | 0.59 | GI_62646802-S | ILMN_59194 | |
| LOC500031 | 1.52 | GI_62646864-S | ILMN_62223 | |
| LOC500048 | 1.64 | GI_62646909-S | ILMN_64357 | |
| LOC500062 | 1.51 | GI_62646942-S | ILMN_65845 | |
| LOC500068 | 1.51 | GI_62646969-S | ILMN_67145 | |
| LOC500229 | 1.62 | GI_62647684-S | ILMN_55274 | |
| LOC500275 | 1.67 | GI_62647889-S | ILMN_65324 | |
| LOC500346 | 1.65 | GI_62648247-S | ILMN_59533 | |
| LOC500359 | 0.53 | GI_62648313-S | ILMN_62675 | |
| LOC500448 | 1.84 | GI_62648877-S | ILMN_68632 | |
| LOC500514 | 3.52 | GI_62649319-S | ILMN_68633 | |
| LOC500537 | 0.63 | GI_62649544-S | ILMN_57485 | |
| LOC500549 | 1.67 | GI_62649688 | ILMN_65156 | |
| LOC500554 | 1.74 | GI_62649724-S | ILMN_66961 | |
| LOC500598 | 0.23 | GI_62650183-S | ILMN_68206 | |
| LOC500707 | 1.73 | GI_62651155-S | ILMN_49697 | |
| LOC500754 | 0.64 | GI_62651582-S | ILMN_48714 | |
| LOC500819 | 1.59 | GI_62651986-S | ILMN_69511 | |
| LOC500824 | 0.55 | GI_62652033-S | ILMN_48958 | |
| LOC500837 | 1.71 | GI_62652190-S | ILMN_56859 | |
| LOC500876 | 0.56 | GI_62652480-S | ILMN_48553 | |
| LOC500892 | 0.66 | GI_34866810-S | ILMN_54102 | |
| LOC500945 | 1.57 | GI_62653242-S | ILMN_65943 | |
| LOC501023 | 1.59 | GI_62653917-S | ILMN_53848 | |
| LOC501040 | 0.64 | GI_62654156-S | ILMN_66043 | |
| LOC501064 | 1.59 | GI_62654411-S | ILMN_55555 | |
| LOC501111 | 1.74 | GI_62654800-S | ILMN_52892 | |
| LOC501112 | 1.75 | GI_62654802-S | ILMN_52983 | |
| LOC501172 | 1.55 | GI_62655278-S | ILMN_54290 | |
| LOC501256 | 4.25 | GI_62717914-S | ILMN_55182 | |
| LOC501312 | 3.21 | GI_62718177-S | ILMN_69417 | |
| LOC501355 | 0.57 | GI_62718302-S | ILMN_52964 | |
| LOC501359 | 0.48 | GI_62718310-S | ILMN_53295 | |
| LOC501415 | 0.32 | GI_62718528-S | ILMN_66501 | |
| LOC501424 | 4.06 | GI_62718566-S | ILMN_68806 | |
| LOC501447 | 0.58 | GI_62718662-S | ILMN_52196 | |
| LOC501502 | 0.52 | GI_62666398-S | ILMN_50570 | |
| LOC501521 | 1.51 | GI_62666551 | ILMN_59239 | |
| LOC501525 | 1.54 | GI_62666613-S | ILMN_63249 | |
| LOC501648 | 1.88 | GI_62667423-S | ILMN_61833 | |
| LOC501649 | 1.8 | GI_62667437-S | ILMN_62720 | |
| LOC501656 | 1.98 | GI_62667501 | ILMN_65898 | |
| LOC501977 | 2.04 | GI_62661255 | ILMN_70056 | |
| LOC502005 | 1.54 | GI_62661940-S | ILMN_69806 | |
| LOC502091 | 2.56 | GI_62662897 | ILMN_53681 | |
| LOC502271 | 1.68 | GI_62638754-S | ILMN_65037 | |
| LOC502490 | 0.49 | GI_62642669-S | ILMN_55350 | |
| LOC502584 | 0.66 | GI_62644035-S | ILMN_56009 | |
| LOC503070 | 1.84 | GI_62651493-S | ILMN_66874 | |
| LOC503119 | 3.5 | GI_62651925-S | ILMN_66511 | |
| LOC503164 | 0.52 | GI_62652776-S | ILMN_63383 | |
| LOC503185 | 2.06 | GI_62653390-S | ILMN_50451 | |
| Loh12cr1_predicted | 1.63 | GI_62648255-S | ILMN_59891 | |
| Lrfn1_predicted | -0.1 | GI_62639246-S | ILMN_68269 | |
| Lrp6_predicted | 1.66 | GI_62648253-S | ILMN_59799 | |
| Lrrc15 | 2.73 | GI_21489966 | ILMN_66084 | |
| Ly49s6 | 1.68 | GI_57222303-S | ILMN_69332 | |
| Ly6g6e | 1.57 | GI_51243042-S | ILMN_58793 | |
| Ly78_predicted | 1.86 | GI_62642846-S | ILMN_63991 | |
| Mafb | 0.59 | GI_31415867-S | ILMN_53361 | |
| Max | 1.52 | GI_11559987-S | ILMN_53697 | |
| Mcpt8l2 | 1.94 | GI_62661509-S | ILMN_69281 | |
| Mei1_predicted | 1.67 | GI_62652809-S | ILMN_64977 | |
| Mfhas1_predicted | 0.64 | GI_34878732-S | ILMN_59565 | |
| Mfrp_predicted | 2.5 | GI_34863202-S | ILMN_59378 | |
| Mlze_predicted | 1.69 | GI_62652495-S | ILMN_49201 | |
| Mocs1_predicted | 1.55 | GI_62654647-S | ILMN_68216 | |
| Mrps10_predicted | 0.45 | GI_57012443-A | ILMN_65339 | |
| Msx1 | 2.16 | GI_13592000-S | ILMN_51890 | |
| Mtmr3_predicted | 1.79 | GI_58865643-A | ILMN_52478 | |
| Myh7b_predicted | 1.55 | GI_62646126-S | ILMN_69497 | |
| Naalad2_predicted | 0.44 | GI_62653273-S | ILMN_67591 | |
| NAPE-PLD | 1.7 | GI_40786460-S | ILMN_50245 | |
| Ncoa5_predicted | 1.52 | GI_62646372-S | ILMN_58832 | |
| Neb_predicted | 1.69 | GI_62645110-S | ILMN_64959 | |
| Nfatc3_predicted | 1.5 | GI_62665192-S | ILMN_59404 | |
| Nfic | 0.4 | GI_62651686-S | ILMN_54364 | |
| Nid | 1.6 | GI_62663699-S | ILMN_63764 | |
| Nkrp2 | 1.74 | GI_19424149-S | ILMN_51410 | |
| Npl4 | 1.57 | GI_18034778-S | ILMN_67824 | |
| Nsddr | 3.12 | GI_54312093-A | ILMN_66303 | |
| Nsg1 | 1.82 | GI_47059499-S | ILMN_61387 | |
| Nup188_predicted | 0.63 | GI_62644862-S | ILMN_52614 | |
| Nxf | 0.62 | GI_24025629-S | ILMN_61837 | |
| Oas1 | 0.33 | GI_25742703-S | ILMN_68688 | |
| Oas1c | 0.56 | GI_57222311-S | ILMN_56594 | |
| Oas1i | 0.46 | GI_57527336-A | ILMN_56232 | |
| Ocm | 0.4 | GI_39930605-S | ILMN_61126 | |
| Odf4 | 0.56 | GI_56090298-S | ILMN_53459 | |
| Olr1199_predicted | 2.67 | GI_47577848-S | ILMN_65648 | |
| Olr1288_predicted | 2.28 | GI_47576286-S | ILMN_52840 | |
| Olr1504 | 0.6 | GI_47575962-S | ILMN_54596 | |
| Olr1553_predicted | 1.54 | GI_47576376-S | ILMN_66920 | |
| Olr1597_predicted | 1.72 | GI_47576458-S | ILMN_52674 | |
| Olr160_predicted | 1.92 | GI_47576484-S | ILMN_49980 | |
| Olr1701_predicted | 1.56 | GI_47576678-S | ILMN_55503 | |
| Olr1714_predicted | 1.55 | GI_47523961-S | ILMN_69741 | |
| Olr1720_predicted | 1.71 | GI_47576704-S | ILMN_53260 | |
| Olr210_predicted | 0.66 | GI_47576854-S | ILMN_48838 | |
| Olr220_predicted | 0.46 | GI_47576874-S | ILMN_61576 | |
| Olr240_predicted | 2.13 | GI_62641101-S | ILMN_69572 | |
| Olr482_predicted | 0.55 | GI_47577488 | ILMN_62817 | |
| Olr488_predicted | 2.11 | GI_47577498-S | ILMN_70082 | |
| Olr557_predicted | 1.58 | GI_47577660-S | ILMN_65470 | |
| Olr693_predicted | 0.58 | GI_47577432-S | ILMN_54407 | |
| Olr753_predicted | 2.06 | GI_47577142-S | ILMN_55711 | |
| Olr789_predicted | 1.68 | GI_47576980-S | ILMN_55239 | |
| Olr818_predicted | 1.58 | GI_47576852-S | ILMN_49483 | |
| Otos | 1.84 | GI_21070937-S | ILMN_67550 | |
| Otx2_predicted | 2.09 | GI_62661288-S | ILMN_50643 | |
| P2rx1 | 0.53 | GI_40254797-S | ILMN_48600 | |
| P34 | 0.54 | GI_19705518-S | ILMN_63435 | |
| Panx1 | 0.25 | GI_40786482-S | ILMN_61939 | |
| Pcca | 3.8 | GI_62662061-S | ILMN_59475 | |
| Pcdh16_predicted | 1.61 | GI_62641085-S | ILMN_68871 | |
| Pcdh18_predicted | 1.57 | GI_62643419-S | ILMN_69675 | |
| Pcdha13 | 0.52 | GI_40789246-S | ILMN_50965 | |
| Pcdhb11_predicted | 0.65 | GI_62339297-S | ILMN_52319 | |
| Pik3c2a_predicted | 1.76 | GI_62641178-S | ILMN_50423 | |
| Pla2g5 | 2.25 | GI_8393973-S | ILMN_58717 | |
| Plac9_predicted | 0.42 | GI_34876899-S | ILMN_52653 | |
| Pld2 | 0.65 | GI_31377515-S | ILMN_56726 | |
| Plekhf2_predicted | 1.53 | GI_62648624-S | ILMN_55541 | |
| Plk1 | 0.63 | GI_25742782-S | ILMN_62013 | |
| Polr3f_predicted | 0.64 | GI_62645963-S | ILMN_61603 | |
| Ppargc1a | 1.79 | GI_13786187-S | ILMN_67033 | |
| Ppm1h_predicted | 1.57 | GI_62652228 | ILMN_58591 | |
| Ppp1r2 | 2.45 | GI_41281690 | ILMN_55618 | |
| Prkaa2 | 1.59 | GI_13027453 | ILMN_56898 | |
| Prkr | 0.64 | GI_9506992-A | ILMN_47830 | |
| Prlph | 1.57 | GI_11024653-S | ILMN_69218 | |
| Prm2 | 0.54 | GI_6981407-S | ILMN_64924 | |
| Prnd_predicted | 0.46 | GI_62645907-S | ILMN_58661 | |
| Ptcd1_predicted | 1.87 | GI_62658435-S | ILMN_50035 | |
| Pter | 1.73 | GI_11560013 | ILMN_52601 | |
| Ptger3 | 1.59 | GI_6981433-S | ILMN_69471 | |
| Ptpn12 | 0.59 | GI_16923959-A | ILMN_51058 | |
| Ptpn14_predicted | 2.82 | GI_62659931-S | ILMN_56142 | |
| Rab31 | 1.51 | GI_21489994-S | ILMN_69477 | |
| Rab3c | 0.67 | GI_19424193-S | ILMN_62518 | |
| Rab3il1 | 0.4 | GI_19705544-S | ILMN_70162 | |
| Rab4b | 1.51 | GI_8394135-S | ILMN_60665 | |
| Rasgrf2 | 4.06 | GI_16758537-S | ILMN_62709 | |
| Rasl10b_predicted | 0.59 | GI_62656675-S | ILMN_62783 | |
| Rem1_predicted | 0.46 | GI_62646063-S | ILMN_66497 | |
| RGD1308915_predicted | 1.56 | GI_62655730 | ILMN_68389 | |
| RGD1309459 | 1.86 | GI_56119163-S | ILMN_68759 | |
| RGD1309487_predicted | 0.62 | GI_62078548-S | ILMN_50173 | |
| RGD1310519_predicted | 0.65 | GI_62078808-S | ILMN_69847 | |
| RGD1311476_predicted | 0.43 | GI_62650205 | ILMN_69256 | |
| Rgl1_predicted | 1.54 | GI_62659480-S | ILMN_50037 | |
| Rgr_predicted | 0.6 | GI_62662268-S | ILMN_59229 | |
| Rgs1 | 1.55 | GI_62659448-S | ILMN_48486 | |
| Rhbdl2_predicted | 2.22 | GI_62649599-S | ILMN_60526 | |
| Rhced | 0.61 | GI_40254767-S | ILMN_50567 | |
| Rin2_predicted | 1.82 | GI_62645978-S | ILMN_62312 | |
| Riok3_predicted | 2.4 | GI_62664046-S | ILMN_53005 | |
| Ripk3 | 1.58 | GI_21326476-S | ILMN_52741 | |
| Rnf151_predicted | 0.45 | GI_62655835-S | ILMN_57140 | |
| Rps6ka2 | 0.6 | GI_62638556-S | ILMN_55518 | |
| Rps9 | 2.42 | GI_13592066-S | ILMN_59654 | |
| RT1-A3 | 0.6 | GI_62665668-A | ILMN_60477 | |
| RT1-CE7 | 0.61 | GI_57012399-A | ILMN_57525 | |
| Rundc1_predicted | 1.64 | GI_62657073-S | ILMN_63936 | |
| Sardh | 1.87 | GI_25742656-S | ILMN_56716 | |
| Sctr | 0.58 | GI_13592080-S | ILMN_56461 | |
| Sema3e_predicted | 1.57 | GI_62646708-S | ILMN_54707 | |
| Senp1_predicted | 2 | GI_62653006-S | ILMN_52116 | |
| Shox2 | 1.59 | GI_6981533-S | ILMN_59659 | |
| Siat8d | 0.65 | GI_62655433-S | ILMN_69350 | |
| Slc22a2 | 0.54 | GI_13994170-S | ILMN_60219 | |
| Slc2a3 | 1.96 | GI_8394300-S | ILMN_56997 | |
| Slc30a2 | 1.51 | GI_6981713-S | ILMN_64418 | |
| Slc35b2 | 3.02 | GI_40018589-S | ILMN_48228 | |
| Slc39a1_predicted | 0.67 | GI_62643809-S | ILMN_66019 | |
| Slc7a8 | 1.74 | GI_16758187-S | ILMN_57185 | |
| Slco1a5 | 2.31 | GI_13540641-S | ILMN_66737 | |
| Snip | 2.05 | GI_9507126-S | ILMN_49321 | |
| Snrk | 1.84 | GI_20301961-A | ILMN_57496 | |
| Sostdc1 | 2.89 | GI_24899634-S | ILMN_63695 | |
| Spag4l_predicted | 1.54 | GI_62646090-S | ILMN_67931 | |
| Stat4_predicted | 0.45 | GI_62655038-S | ILMN_52470 | |
| Stat5b | 0.58 | GI_11693139-S | ILMN_53449 | |
| Steap_predicted | 1.73 | GI_62646763-S | ILMN_57381 | |
| Strn | 0.64 | GI_62650226-A | ILMN_49273 | |
| Susd2_predicted | 1.95 | GI_62665860-S | ILMN_69371 | |
| Synpo | 1.6 | GI_11067428-S | ILMN_59961 | |
| Taar3 | 1.62 | GI_57222339-S | ILMN_68098 | |
| Tacstd2 | 0.36 | GI_57164020-S | ILMN_55110 | |
| Tas2r7 | 1.62 | GI_13027466-S | ILMN_51499 | |
| Tbpl1_predicted | 0.45 | GI_62638383-S | ILMN_69666 | |
| Tbx2_predicted | 1.86 | GI_62656827-S | ILMN_47842 | |
| Tcerg1_predicted | 3.61 | GI_62664334-S | ILMN_62175 | |
| Tead2 | 1.51 | GI_62639723-S | ILMN_63523 | |
| Terc | 0.43 | GI_34850062-S | ILMN_57998 | |
| Tgm1 | 2.21 | GI_13928911-S | ILMN_61579 | |
| Thbs4 | 1.5 | GI_62642736-S | ILMN_58654 | |
| Thrap2_predicted | 0.55 | GI_62658968-S | ILMN_58605 | |
| Tigd3_predicted | 3.21 | GI_62641770-S | ILMN_57464 | |
| Tm7sf1_predicted | 1.62 | GI_62663701-S | ILMN_63856 | |
| Tmc4_predicted | 1.68 | GI_62638819-S | ILMN_68268 | |
| Tmem27 | 3.38 | GI_10198601-S | ILMN_55252 | |
| Tna_predicted | 1.64 | GI_62654554-S | ILMN_62956 | |
| Tnrc6_predicted | 0.58 | GI_62641248-S | ILMN_53904 | |
| Top3a_predicted | 1.54 | GI_62656176-S | ILMN_52551 | |
| Trdn | 1.54 | GI_31340608-S | ILMN_61674 | |
| Trim14_predicted | 1.71 | GI_62649065-S | ILMN_55189 | |
| Trim63 | 1.63 | GI_18266713-S | ILMN_55817 | |
| Trpc6 | 1.8 | GI_16758329-S | ILMN_69192 | |
| Trpv4 | 1.53 | GI_13027423-S | ILMN_68220 | |
| Ttr | 2.2 | GI_6981683-S | ILMN_53794 | |
| Tubal3_predicted | 1.77 | GI_62663791-S | ILMN_68925 | |
| Tubd1_predicted | 0.52 | GI_62656760-S | ILMN_67083 | |
| Unc5d_predicted | 1.56 | GI_62662798-S | ILMN_47954 | |
| Ung_predicted | 1.57 | GI_61557030-S | ILMN_62789 | |
| Usf2 | 1.6 | GI_13592124-S | ILMN_54156 | |
| Usp24_predicted | 0.37 | GI_62649323-S | ILMN_68810 | |
| Vax1 | 4.35 | GI_12018333-S | ILMN_57171 | |
| Vax2 | 1.73 | GI_12018335-S | ILMN_52425 | |
| Vof16 | 3.02 | GI_22219439-S | ILMN_57985 | |
| Wnt5a | 0.52 | GI_12018323-S | ILMN_57730 | |
| Zfhx1b_predicted | 1.84 | GI_62645070-S | ILMN_63021 | |
| Zfhx2 | 0.65 | GI_41054881-S | ILMN_55713 | |
| Zfp212_predicted | 1.51 | GI_62647195-S | ILMN_55087 | |
| Zfp239_predicted | 0.65 | GI_62664119 | ILMN_64483 | |
| Zfp287_predicted | 1.59 | GI_62656218-S | ILMN_54828 | |
| Zfp364_predicted | 0.51 | GI_62643934-S | ILMN_49587 | |
| Zfp509_predicted | 0.65 | GI_62660681-S | ILMN_67270 | |
| Zfp54_predicted | 1.62 | GI_62638784-S | ILMN_66668 | |
| Zfp597 | 0.65 | GI_24308495-S | ILMN_54677 | |
| Zhx2 | 0.55 | GI_62652443-S | ILMN_69512 | |
| Zmynd15_predicted | 2.87 | GI_62656360-S | ILMN_62337 | |
| Znf183 | 0.53 | GI_52138730-S | ILMN_58247 | |
| Znf354c | 1.81 | GI_13027447-S | ILMN_68591 | |
|  | 1.59 | Rn.8739 | ILMN_161251 | |
|  | 0.61 | Rn.3659 | ILMN_161556 | |
