# Supplementary material for: Effects of Xiaoyaosan on the Hippocampal Gene Expression Profile in Rats Subjected to Chronic Immobilization Stress
Source: Front Psychiatry. 2019 Apr 12;10:178. doi: 10.3389/fpsyt.2019.00178 (PMC6474260; doi:10.3389/fpsyt.2019.00178)
Supplement: Supplementary file 2 [file Table_2.docx]

Table 2 Differentially expressed gene in the XYS group compared with the model group

| SYMBOL | Ratio(21T/21M) | SEARCH_KEY | TargetID |
| --- | --- | --- | --- |
| Acaca | 0.45 | GI_11559961-S | ILMN_49001 |
| Adamts16_predicted | 1.51 | GI_62663085-S | ILMN_56605 |
| Agpt2 | 1.64 | GI_62662893 | ILMN_53507 |
| Aim1_predicted | 0.58 | GI_62666321-S | ILMN_68843 |
| Alox15 | 1.81 | GI_31542124-S | ILMN_67647 |
| Anapc2_predicted | 1.57 | GI_62644719-S | ILMN_68445 |
| Ang1 | 0.63 | GI_62661280-A | ILMN_50295 |
| Apoa5 | 1.57 | GI_18034776-S | ILMN_51589 |
| Apobec1 | 0.59 | GI_55742830-S | ILMN_57819 |
| Aqp1 | 0.66 | GI_6978526-S | ILMN_53187 |
| Arhgef7 | 14.4 | GI_16758563-S | ILMN_53958 |
| Arl11_predicted | 1.55 | GI_34875166 | ILMN_48311 |
| Arrb2 | 0.64 | GI_6978538-S | ILMN_56088 |
| Asmt | 2.19 | GI_21426784-S | ILMN_63771 |
| Atp7a | 2.79 | GI_16258816-S | ILMN_53897 |
| Atp8b2_predicted | 1.56 | GI_62643803-S | ILMN_65755 |
| Blk_predicted | 1.84 | GI_62661615-S | ILMN_52137 |
| C1qtnf1 | 1.74 | GI_56090623-S | ILMN_52439 |
| C5 | 1.62 | GI_62645000-S | ILMN_59614 |
| Cdc2a | 1.69 | GI_9506474-S | ILMN_58988 |
| Cdc42ep5_predicted | 0.65 | GI_62638903-S | ILMN_49668 |
| Cdh22 | 0.08 | GI_9506476-S | ILMN_50959 |
| Cdk6 | 1.71 | GI_62646796-S | ILMN_58924 |
| Cdkl3 | 0.6 | GI_13489070-S | ILMN_66469 |
| Cdkn3_predicted | 0.66 | GI_62661258-S | ILMN_48042 |
| Chrng | 0.4 | GI_9506488-S | ILMN_52509 |
| Cldn1 | 0.55 | GI_13928975 | ILMN_49581 |
| Cldn4_predicted | 0.4 | GI_62658763-A | ILMN_67869 |
| Clic4 | 1.53 | GI_13929165-S | ILMN_69999 |
| Cnot6l_predicted | 1.64 | GI_62660189-S | ILMN_53235 |
| Cnr1 | 0.66 | GI_52421334-S | ILMN_51681 |
| Cntfr | 1.57 | GI_51556266-S | ILMN_60678 |
| Col14a1_predicted | 1.85 | GI_62652431-S | ILMN_68990 |
| Col1a1 | 1.59 | GI_62656859-S | ILMN_50637 |
| Col8a1_predicted | 0.46 | GI_62657772-S | ILMN_70226 |
| Cpz | 1.52 | GI_13929065-S | ILMN_57440 |
| Crhr2 | 1.59 | GI_12083686-S | ILMN_58814 |
| Cryba2 | 0.57 | GI_27465610-S | ILMN_63872 |
| Ctns_predicted | 0.52 | GI_62656510-S | ILMN_53493 |
| Cutl2_predicted | 1.68 | GI_62658913-S | ILMN_54928 |
| Cxcl11 | 95.67 | GI_54262189-S | ILMN_55704 |
| Cxcl9 | 1.64 | GI_56799386-S | ILMN_69223 |
| Cyp2a2 | 1.52 | GI_6978740-S | ILMN_59498 |
| Cyp4b1 | 3.1 | GI_23397410-S | ILMN_57699 |
| Dbh | 1.94 | GI_25742779-S | ILMN_66537 |
| Depdc2_predicted | 0.65 | GI_62648534-S | ILMN_51440 |
| Dll3 | 0.59 | GI_16758479-S | ILMN_68661 |
| Dmrta2_predicted | 0.44 | GI_62649413-S | ILMN_50442 |
| Dnajc8_predicted | 0.35 | GI_34871853 | ILMN_48107 |
| Dnase1l2_predicted | 0.65 | GI_62655824-S | ILMN_56503 |
| Dntt_predicted | 0.16 | GI_60223046-S | ILMN_56193 |
| Doxl2 | 1.62 | GI_40556303-S | ILMN_66661 |
| Dpysl3 | 0.67 | GI_25742567-S | ILMN_59958 |
| Dtr | 0.57 | GI_6978784-S | ILMN_51692 |
| Edg7_predicted | 0.66 | GI_62644500-S | ILMN_56476 |
| Efna1 | 0.44 | GI_16758383 | ILMN_59597 |
| Efna2 | 0.66 | GI_62651931-S | ILMN_66783 |
| Enpp3 | 0.67 | GI_62638345-A | ILMN_67825 |
| Enpp6_predicted | 1.51 | GI_62662655-S | ILMN_50650 |
| Ephb3_predicted | 0.59 | GI_62658140-S | ILMN_52730 |
| Eraf_predicted | 1.6 | GI_62641362-S | ILMN_59605 |
| Erbb4 | 0.61 | GI_11067414-S | ILMN_58425 |
| Esrrb | 0.44 | GI_56606091-S | ILMN_55858 |
| Evx2_predicted | 1.67 | GI_62645254-S | ILMN_49590 |
| F5 | 0.58 | GI_62659616-S | ILMN_59469 |
| Fabp1 | 0.66 | GI_6978824-S | ILMN_56910 |
| Fam31b_predicted | 0.61 | GI_62659419-S | ILMN_69801 |
| Fanca_predicted | 4.39 | GI_62665410-S | ILMN_70242 |
| Fancc | 0.51 | GI_6978828-S | ILMN_54510 |
| Fgf14 | 0.43 | GI_19224656-S | ILMN_63613 |
| Figf | 1.69 | GI_13929073-S | ILMN_50127 |
| Folr2_predicted | 0.65 | GI_62641037-S | ILMN_66486 |
| Foxa3 | 0.5 | GI_8393540 | ILMN_51858 |
| Frs2_predicted | 1.75 | GI_62652128-S | ILMN_53397 |
| Ftmt_predicted | 1.93 | GI_62664413-S | ILMN_49805 |
| Fxyd2 | 1.61 | GI_22538396-I | ILMN_67725 |
| Fyb_predicted | 0.38 | GI_62643003-S | ILMN_49585 |
| Gabra1 | 0.56 | GI_34328531-S | ILMN_53178 |
| Gas8_predicted | 1.52 | GI_62665425-S | ILMN_48237 |
| Gja9 | 0.62 | GI_9506728-S | ILMN_47873 |
| Gje1 | 0.48 | GI_34871468-S | ILMN_61454 |
| Glis1_predicted | 0.64 | GI_62649379-S | ILMN_48869 |
| Glp2r | 3.07 | GI_11177881-S | ILMN_59344 |
| Gnat2_predicted | 3.03 | GI_62644176-S | ILMN_63019 |
| Gpnmb | 1.66 | GI_18959233-S | ILMN_67552 |
| Gpr103 | 0.57 | GI_38016149-S | ILMN_52251 |
| Gpr30 | 0.57 | GI_19424261-S | ILMN_63610 |
| Gpr31_predicted | 2.42 | GI_27730160-S | ILMN_57736 |
| Gpr40 | 1.75 | GI_23463290-S | ILMN_58187 |
| Gpr45_predicted | 1.79 | GI_62654975-S | ILMN_65864 |
| Gpx2 | 0.62 | GI_34577115-S | ILMN_65301 |
| Grb2 | 2.03 | GI_13540653-S | ILMN_69214 |
| Grid1 | 2.1 | GI_13259379-S | ILMN_53503 |
| Guk1_predicted | 1.59 | GI_62656143 | ILMN_50547 |
| H2a | 0.6 | GI_62663548 | ILMN_55583 |
| Hao1 | 1.52 | GI_62645919-S | ILMN_59192 |
| Hcst | 2.07 | GI_62639284-S | ILMN_70266 |
| Hdac1 | 1.52 | GI_62655264-I | ILMN_53227 |
| Hemgn | 1.51 | GI_19882214-S | ILMN_59672 |
| Hspcb | 1.73 | GI_51890228-S | ILMN_49991 |
| Ibsp | 0.65 | GI_6981065-S | ILMN_56630 |
| Iiig9 | 0.4 | GI_21955165-S | ILMN_52168 |
| Il22ra2 | 2.23 | GI_50979277-S | ILMN_60050 |
| Il23a | 1.54 | GI_18426831-S | ILMN_58716 |
| Irs1 | 1.51 | GI_6981105-S | ILMN_70074 |
| Ka17 | 0.6 | GI_47087084-S | ILMN_62108 |
| Kb9 | 1.93 | GI_57012371-S | ILMN_63509 |
| Kcna1 | 0.66 | GI_31377502-S | ILMN_58093 |
| Kcnj8 | 0.65 | GI_51036640-S | ILMN_69136 |
| Kif12_predicted | 1.53 | GI_62649058-S | ILMN_54810 |
| Kif26a_predicted | 0.46 | GI_62651289-S | ILMN_56213 |
| Krt1-12_predicted | 0.52 | GI_56912238-A | ILMN_58498 |
| Kua_predicted | 1.54 | Rn.101810 | ILMN_160579 |
| Lad1_predicted | 1.51 | GI_62659370-S | ILMN_64473 |
| Lias_predicted | 1.63 | GI_62660459-S | ILMN_49136 |
| Liph_predicted | 0.66 | GI_62657709-S | ILMN_63843 |
| LOC171553 | 1.94 | GI_40254730-S | ILMN_67904 |
| LOC191574 | 1.54 | GI_19924086-A | ILMN_56158 |
| LOC286986 | 1.62 | GI_29336098-S | ILMN_57632 |
| LOC287044 | 1.51 | GI_62655667-S | ILMN_65343 |
| LOC287101 | 1.52 | GI_62655759-S | ILMN_69876 |
| LOC287157 | 0.44 | GI_62655890-S | ILMN_60262 |
| LOC287612 | 1.59 | GI_62656748 | ILMN_66524 |
| LOC287708 | 1.56 | GI_62643229-S | ILMN_60507 |
| LOC287881 | 1.6 | GI_27690901-S | ILMN_161773 |
| LOC288044 | 0.58 | GI_62658007-S | ILMN_66054 |
| LOC288272 | 0.42 | GI_62657614-S | ILMN_59035 |
| LOC288485 | 2.38 | GI_62658473-S | ILMN_51946 |
| LOC288907 | 1.78 | GI_62665085-S | ILMN_53600 |
| LOC289052 | 1.49 | GI_62659423-S | ILMN_69963 |
| LOC289094 | 0.66 | GI_62659510-S | ILMN_51465 |
| LOC289245 | 0.62 | GI_62659826-S | ILMN_48304 |
| LOC289334 | 0.63 | GI_62659883-S | ILMN_53591 |
| LOC289529 | 1.78 | GI_27694190-S | ILMN_161780 |
| LOC290489 | 0.66 | GI_62662067-S | ILMN_59740 |
| LOC290500 | 2 | GI_62662063-S | ILMN_59563 |
| LOC290678 | 0.29 | GI_62662494-S | ILMN_54661 |
| LOC290823 | 0.43 | GI_62662821-S | ILMN_49142 |
| LOC291034 | 0.52 | GI_62663392-S | ILMN_51963 |
| LOC291958 | 0.59 | GI_62665140-S | ILMN_56611 |
| LOC292151 | 1.7 | GI_62642560-S | ILMN_70271 |
| LOC292690 | 0.53 | GI_62639104-S | ILMN_60780 |
| LOC292847 | 2.19 | GI_62639610-S | ILMN_59426 |
| LOC293310 | 0.52 | GI_62641432-S | ILMN_63013 |
| LOC293864 | 1.71 | GI_62667556-S | ILMN_68846 |
| LOC294375 | 0.62 | GI_62666056-S | ILMN_55974 |
| LOC294722 | 0.62 | GI_27687434-S | ILMN_48361 |
| LOC295047 | 2.07 | GI_62643456-S | ILMN_48696 |
| LOC295090 | 0.62 | GI_62643518-S | ILMN_51427 |
| LOC295352 | 1.76 | GI_62644346-S | ILMN_48779 |
| LOC295356 | 0.58 | GI_62078556-S | ILMN_62309 |
| LOC296129 | 0.58 | GI_62646138-S | ILMN_70020 |
| LOC296778 | 2 | GI_62718193-S | ILMN_70111 |
| LOC296782 | 1.84 | GI_62664983-S | ILMN_48499 |
| LOC297444 | 1.63 | GI_62647794-S | ILMN_60800 |
| LOC297805 | 0.45 | GI_62648588-S | ILMN_53924 |
| LOC297890 | 0.51 | GI_62649722-S | ILMN_66870 |
| LOC297892 | 0.65 | GI_27732752-S | ILMN_67409 |
| LOC298014 | 0.64 | GI_62649005-S | ILMN_52369 |
| LOC298493 | 1.6 | GI_34870900-S | ILMN_58398 |
| LOC300108 | 2 | GI_62652961 | ILMN_49856 |
| LOC300120 | 0.66 | GI_62652873-S | ILMN_68300 |
| LOC300742 | 0.61 | GI_62653735-S | ILMN_68051 |
| LOC301378 | 1.51 | GI_62654979-S | ILMN_66425 |
| LOC301518 | 1.8 | GI_27684996-S | ILMN_161763 |
| LOC301628 | 2.06 | GI_62655467-S | ILMN_48478 |
| LOC301742 | 1.84 | GI_62646980-S | ILMN_67666 |
| LOC302264 | 0.36 | GI_62718151-S | ILMN_68286 |
| LOC302278 | 1.51 | GI_62664975 | ILMN_48145 |
| LOC302325 | 2.26 | GI_27675137-S | ILMN_49150 |
| LOC302552 | 0.54 | GI_34933406-S | ILMN_61740 |
| LOC302809 | 0.48 | GI_62667359-S | ILMN_58166 |
| LOC303542 | 0.59 | GI_62657054-S | ILMN_62961 |
| LOC303575 | 0.11 | GI_62657157-S | ILMN_69523 |
| LOC303660 | 0.66 | GI_62657305-S | ILMN_56775 |
| LOC303677 | 1.84 | GI_62657349-S | ILMN_59218 |
| LOC304318 | 1.63 | GI_62658510-S | ILMN_54469 |
| LOC304530 | 0.6 | GI_62659024-S | ILMN_61455 |
| LOC304575 | 0.6 | GI_62659090-S | ILMN_65525 |
| LOC304650 | 0.62 | GI_62665036-S | ILMN_51022 |
| LOC305448 | 1.83 | GI_62660640-S | ILMN_65091 |
| LOC305844 | 1.9 | GI_62661366-S | ILMN_54842 |
| LOC306504 | 0.57 | GI_62662718-S | ILMN_54753 |
| LOC306511 | 1.66 | GI_62662778 | ILMN_61196 |
| LOC307855 | 1.59 | GI_62665293-S | ILMN_64668 |
| LOC307989 | 0.59 | GI_62642526-S | ILMN_68699 |
| LOC308724 | 1.5 | GI_62640724-S | ILMN_51328 |
| LOC308894 | 1.58 | GI_62641156-S | ILMN_49418 |
| LOC308990 | 0.62 | GI_62641303-S | ILMN_56832 |
| LOC309100 | 0.57 | GI_62641582-S | ILMN_70269 |
| LOC309357 | 0.55 | GI_62667512-S | ILMN_66553 |
| LOC310212 | 1.51 | GI_34855068-S | ILMN_58655 |
| LOC310250 | 0.56 | GI_62643214-S | ILMN_59698 |
| LOC310596 | 0.61 | GI_34858190-S | ILMN_52612 |
| LOC311171 | 0.27 | GI_62645359-S | ILMN_54892 |
| LOC311352 | 0.63 | GI_62645662 | ILMN_69680 |
| LOC311861 | 0.62 | GI_62644924-S | ILMN_55919 |
| LOC312490 | 2.27 | GI_62647693-S | ILMN_55841 |
| LOC312677 | 0.51 | GI_62648099-S | ILMN_52197 |
| LOC312683 | 1.55 | GI_62648112-S | ILMN_52792 |
| LOC313609 | 0.67 | GI_62649824-S | ILMN_49439 |
| LOC313906 | 0.4 | GI_62650390-S | ILMN_57217 |
| LOC314119 | 2.57 | GI_62666934-S | ILMN_56894 |
| LOC314785 | 0.54 | GI_62652058-S | ILMN_50107 |
| LOC314858 | 0.36 | GI_62652179-S | ILMN_56215 |
| LOC314927 | 1.57 | GI_62652358-S | ILMN_65246 |
| LOC315069 | 0.66 | GI_62652561-S | ILMN_52296 |
| LOC315130 | 1.9 | GI_62652725-S | ILMN_60903 |
| LOC315423 | 0.64 | GI_62653229-S | ILMN_65336 |
| LOC315465 | 2.45 | GI_34860442-S | ILMN_47930 |
| LOC315728 | 0.34 | GI_62653981-S | ILMN_57136 |
| LOC315833 | 1.92 | GI_27720832-S | ILMN_54105 |
| LOC316122 | 0.55 | GI_47058977-S | ILMN_51978 |
| LOC317168 | 0.66 | GI_62664390-S | ILMN_48577 |
| LOC317227 | 0.51 | GI_62667102-S | ILMN_66644 |
| LOC317398 | 1.54 | GI_62666726-S | ILMN_69026 |
| LOC317420 | 0.61 | GI_62666738-S | ILMN_69635 |
| LOC317423 | 0.64 | GI_62666740-S | ILMN_69728 |
| LOC360425 | 0.55 | GI_62652542-S | ILMN_51356 |
| LOC360527 | 0.66 | GI_62656081-S | ILMN_70223 |
| LOC360589 | 0.6 | GI_62656720-S | ILMN_64988 |
| LOC360840 | 0.59 | GI_62659285-S | ILMN_58776 |
| LOC360885 | 0.42 | GI_62659847-S | ILMN_51656 |
| LOC361034 | 0.5 | GI_62661338-S | ILMN_53158 |
| LOC361120 | 1.61 | GI_62662371-S | ILMN_48404 |
| LOC361277 | 0.35 | GI_62663909-S | ILMN_60745 |
| LOC361401 | 0.56 | GI_62665091-A | ILMN_54041 |
| LOC361466 | 0.65 | GI_62638423-S | ILMN_48847 |
| LOC361548 | 0.63 | GI_62639376-S | ILMN_52176 |
| LOC361582 | 0.66 | GI_62639820-S | ILMN_66485 |
| LOC361780 | 0.42 | GI_62642612-S | ILMN_51711 |
| LOC361911 | 0.59 | GI_62643127-S | ILMN_55439 |
| LOC361914 | 1.66 | GI_62643151-S | ILMN_56557 |
| LOC362083 | 0.56 | GI_62644745-S | ILMN_69678 |
| LOC362121 | 0.62 | GI_62645006-S | ILMN_59977 |
| LOC362236 | 0.35 | GI_62646044 | ILMN_65586 |
| LOC362265 | 1.71 | GI_62646319-S | ILMN_55751 |
| LOC362437 | 1.68 | GI_62648164-S | ILMN_55453 |
| LOC362573 | 1.54 | GI_62649527-S | ILMN_56490 |
| LOC362702 | 1.75 | GI_62650361-S | ILMN_55651 |
| LOC362801 | 1.93 | GI_62651580-S | ILMN_48630 |
| LOC362832 | 0.55 | GI_62651728-S | ILMN_56579 |
| LOC363030 | 1.57 | GI_62653344-S | ILMN_48289 |
| LOC363031 | 1.63 | GI_62653346-S | ILMN_48386 |
| LOC363057 | 0.63 | GI_34863327-S | ILMN_62152 |
| LOC363397 | 1.65 | GI_62718354-S | ILMN_55273 |
| LOC363398 | 3.51 | GI_62718283-S | ILMN_52102 |
| LOC363408 | 3.92 | GI_62718656-S | ILMN_51921 |
| LOC363507 | 0.64 | GI_62667435-S | ILMN_62533 |
| LOC363536 | 0.43 | GI_62655622-S | ILMN_63222 |
| LOC363844 | 0.62 | GI_34870025-S | ILMN_60916 |
| LOC363865 | 5.79 | GI_62658398-S | ILMN_70399 |
| LOC364082 | 0.64 | GI_62659994-S | ILMN_61008 |
| LOC364102 | 0.63 | GI_62660129-S | ILMN_48975 |
| LOC364379 | 1.66 | GI_62177151-S | ILMN_64657 |
| LOC364495 | 0.59 | GI_62661908-S | ILMN_67784 |
| LOC364653 | 0.57 | GI_62663044-S | ILMN_69537 |
| LOC364899 | 2 | GI_62664631-S | ILMN_51766 |
| LOC365242 | 0.38 | GI_62639749-S | ILMN_64251 |
| LOC365297 | 0.57 | GI_62640790-S | ILMN_54338 |
| LOC365672 | 1.71 | GI_34854235-S | ILMN_48695 |
| LOC365813 | 0.46 | GI_62643555-S | ILMN_53117 |
| LOC365987 | 0.63 | GI_62644663-S | ILMN_65583 |
| LOC366078 | 1.82 | GI_62645271-S | ILMN_50518 |
| LOC366137 | 1.86 | GI_62645447-S | ILMN_59358 |
| LOC366254 | 0.46 | GI_62646426-S | ILMN_61691 |
| LOC366498 | 1.72 | GI_62650119-S | ILMN_64276 |
| LOC366523 | 2.5 | GI_62650216-S | ILMN_48200 |
| LOC366604 | 0.59 | GI_62650630 | ILMN_69337 |
| LOC366619 | 1.61 | GI_62650673-S | ILMN_48628 |
| LOC366669 | 0.66 | GI_62650894-S | ILMN_59717 |
| LOC366680 | 0.66 | GI_62650931 | ILMN_61703 |
| LOC366768 | 1.64 | GI_62651553-S | ILMN_69597 |
| LOC367539 | 1.58 | GI_62718419-S | ILMN_58927 |
| LOC367860 | 0.32 | GI_62667086-S | ILMN_65730 |
| LOC367994 | 1.63 | GI_62658718-S | ILMN_65176 |
| LOC497687 | 0.29 | GI_62659036-S | ILMN_62608 |
| LOC497694 | 1.76 | GI_62660095-S | ILMN_70142 |
| LOC497717 | 0.52 | GI_62664009-S | ILMN_51192 |
| LOC497746 | 0.55 | GI_62665906-S | ILMN_49071 |
| LOC497757 | 0.6 | GI_62643665-S | ILMN_58917 |
| LOC497821 | 1.83 | GI_62652254-S | ILMN_60166 |
| LOC497830 | 1.62 | GI_62653430-S | ILMN_52720 |
| LOC497917 | 1.5 | GI_62656178-S | ILMN_52643 |
| LOC497918 | 0.65 | GI_62656180-S | ILMN_52728 |
| LOC497972 | 1.73 | GI_62656688-S | ILMN_63478 |
| LOC498007 | 0.64 | GI_62657161 | ILMN_69705 |
| LOC498055 | 0.6 | GI_62657602-S | ILMN_58500 |
| LOC498077 | 0.62 | GI_62657860-S | ILMN_58141 |
| LOC498221 | 1.68 | GI_62659238-S | ILMN_55480 |
| LOC498239 | 1.85 | GI_62659417-S | ILMN_69710 |
| LOC498258 | 0.28 | GI_62659560-S | ILMN_56595 |
| LOC498292 | 0.54 | GI_62659790-S | ILMN_69358 |
| LOC498335 | 2.37 | GI_62660191-S | ILMN_53325 |
| LOC498340 | 0.63 | GI_62660298-S | ILMN_61543 |
| LOC498356 | 2.07 | GI_62660433-S | ILMN_70403 |
| LOC498365 | 1.66 | GI_62660472 | ILMN_49795 |
| LOC498439 | 1.64 | GI_62661012-S | ILMN_51660 |
| LOC498451 | 2.01 | GI_62661116-S | ILMN_60371 |
| LOC498522 | 0.65 | GI_62661542-S | ILMN_48402 |
| LOC498587 | 0.64 | GI_62662250-S | ILMN_58244 |
| LOC498604 | 1.76 | GI_62662401-S | ILMN_49722 |
| LOC498625 | 2.09 | GI_62662605-S | ILMN_64750 |
| LOC498637 | 1.61 | GI_62662708-S | ILMN_54126 |
| LOC498706 | 0.6 | GI_62663236-S | ILMN_47860 |
| LOC498738 | 1.75 | GI_62663472-S | ILMN_56979 |
| LOC498747 | 0.44 | GI_62663512-S | ILMN_59400 |
| LOC498950 | 3.21 | GI_62665268-S | ILMN_63330 |
| LOC498972 | 0.49 | GI_62665513-S | ILMN_52574 |
| LOC499056 | 0.51 | GI_62638790-S | ILMN_66936 |
| LOC499083 | 1.63 | GI_62638936-S | ILMN_51419 |
| LOC499162 | 0.6 | GI_62639827-S | ILMN_66754 |
| LOC499222 | 0.56 | GI_62641065-S | ILMN_67920 |
| LOC499242 | 2.2 | GI_62641126-S | ILMN_48087 |
| LOC499334 | 0.65 | GI_62642037-S | ILMN_48264 |
| LOC499350 | 0.4 | GI_62642219-S | ILMN_54167 |
| LOC499476 | 0.57 | GI_62666311-S | ILMN_68411 |
| LOC499781 | 0.66 | GI_62644952-S | ILMN_57290 |
| LOC499792 | 0.51 | GI_62645052-S | ILMN_62132 |
| LOC499818 | 0.66 | GI_62645325-S | ILMN_53289 |
| LOC499855 | 1.61 | GI_62645518-S | ILMN_62934 |
| LOC499865 | 0.49 | GI_62645568-S | ILMN_65229 |
| LOC499872 | 1.61 | GI_62645615-S | ILMN_67484 |
| LOC499933 | 0.59 | GI_62646233-S | ILMN_51808 |
| LOC499985 | 1.75 | GI_62646700-S | ILMN_54350 |
| LOC500048 | 0.51 | GI_62646909-S | ILMN_64357 |
| LOC500245 | 0.51 | GI_62647751-S | ILMN_58837 |
| LOC500316 | 1.56 | GI_62648156-S | ILMN_55091 |
| LOC500355 | 1.67 | GI_62648295-S | ILMN_61877 |
| LOC500464 | 0.64 | GI_62649015-S | ILMN_52878 |
| LOC500474 | 0.6 | GI_62649052-S | ILMN_54536 |
| LOC500484 | 1.63 | GI_62649136-S | ILMN_59201 |
| LOC500514 | 0.27 | GI_62649319-S | ILMN_68633 |
| LOC500537 | 2.4 | GI_62649544-S | ILMN_57485 |
| LOC500549 | 0.59 | GI_62649688 | ILMN_65156 |
| LOC500565 | 1.57 | GI_62649834-S | ILMN_49932 |
| LOC500718 | 3.95 | GI_62651245-S | ILMN_54099 |
| LOC500724 | 0.53 | GI_62651272 | ILMN_55372 |
| LOC500760 | 0.63 | GI_62651633-S | ILMN_51545 |
| LOC500853 | 0.45 | GI_62652316-S | ILMN_63301 |
| LOC500854 | 0.65 | GI_62652326-S | ILMN_63737 |
| LOC500940 | 1.93 | GI_62653186-S | ILMN_62953 |
| LOC500945 | 0.63 | GI_62653242-S | ILMN_65943 |
| LOC500986 | 0.65 | GI_62653574-S | ILMN_59811 |
| LOC501005 | 2.1 | GI_62653737-S | ILMN_68134 |
| LOC501027 | 0.66 | GI_62654039-S | ILMN_60171 |
| LOC501057 | 1.65 | GI_62654345-S | ILMN_52544 |
| LOC501059 | 1.66 | GI_62654360-S | ILMN_53224 |
| LOC501088 | 0.59 | GI_62654622-S | ILMN_67075 |
| LOC501104 | 1.76 | GI_62654727-S | ILMN_49449 |
| LOC501112 | 0.52 | GI_62654802-S | ILMN_52983 |
| LOC501174 | 0.62 | GI_62655295-S | ILMN_55382 |
| LOC501256 | 0.38 | GI_62717914-S | ILMN_55182 |
| LOC501275 | 0.58 | GI_62718000-S | ILMN_60153 |
| LOC501308 | 0.56 | GI_62718167-S | ILMN_68977 |
| LOC501312 | 0.57 | GI_62718177-S | ILMN_69417 |
| LOC501315 | 1.62 | GI_62718185-S | ILMN_69771 |
| LOC501329 | 1.63 | GI_62718225-S | ILMN_49112 |
| LOC501355 | 1.68 | GI_62718302-S | ILMN_52964 |
| LOC501357 | 1.63 | GI_62718306-S | ILMN_53126 |
| LOC501362 | 2.01 | GI_62718320-S | ILMN_53744 |
| LOC501370 | 1.68 | GI_62718338-S | ILMN_54532 |
| LOC501406 | 2.13 | GI_62718506-S | ILMN_65151 |
| LOC501415 | 3.63 | GI_62718528-S | ILMN_66501 |
| LOC501424 | 0.45 | GI_62718566-S | ILMN_68806 |
| LOC501447 | 1.52 | GI_62718662-S | ILMN_52196 |
| LOC501451 | 0.51 | GI_62718697-S | ILMN_54623 |
| LOC501469 | 1.5 | GI_62718764-S | ILMN_60072 |
| LOC501502 | 2.17 | GI_62666398-S | ILMN_50570 |
| LOC501530 | 1.65 | GI_62666655-S | ILMN_65548 |
| LOC501572 | 1.79 | GI_62666902-S | ILMN_55230 |
| LOC501605 | 1.53 | GI_62667132-S | ILMN_68333 |
| LOC501610 | 2.4 | GI_62667156 | ILMN_69463 |
| LOC501648 | 0.44 | GI_62667423-S | ILMN_61833 |
| LOC501656 | 0.64 | GI_62667501 | ILMN_65898 |
| LOC501786 | 0.55 | GI_62658259-S | ILMN_58603 |
| LOC501949 | 1.9 | GI_62661076-S | ILMN_56689 |
| LOC502091 | 0.4 | GI_62662897 | ILMN_53681 |
| LOC502118 | 2.01 | GI_62663355-S | ILMN_60836 |
| LOC502145 | 2.68 | GI_62663873-S | ILMN_51575 |
| LOC502262 | 1.54 | GI_62638730-S | ILMN_63797 |
| LOC502292 | 0.31 | GI_62638997-S | ILMN_54514 |
| LOC502490 | 0.61 | GI_62642669-S | ILMN_55350 |
| LOC502794 | 1.49 | GI_62647517-S | ILMN_70374 |
| LOC502827 | 1.92 | GI_62647576-S | ILMN_50182 |
| LOC502840 | 0.34 | GI_62647598-S | ILMN_51152 |
| LOC503119 | 0.42 | GI_62651925-S | ILMN_66511 |
| LOC503214 | 0.61 | GI_62653995-S | ILMN_57855 |
| LOC503264 | 0.44 | GI_62655315-S | ILMN_56771 |
| LOC503275 | 1.66 | GI_62655556-S | ILMN_54919 |
| LOC503279 | 0.66 | GI_62717720-S | ILMN_67234 |
| LOC503414 | 3.83 | GI_62718772-S | ILMN_60980 |
| LOC503465 | 2.62 | GI_62667264-S | ILMN_52923 |
| Lrrc15 | 0.31 | GI_21489966 | ILMN_66084 |
| Lrrc8_predicted | 0.57 | GI_62644859-S | ILMN_52444 |
| Ltbr | 1.51 | GI_56605709-S | ILMN_50310 |
| Ly49i8 | 1.6 | GI_57222299-S | ILMN_70114 |
| Ly49s6 | 0.57 | GI_57222303-S | ILMN_69332 |
| Ly78_predicted | 0.66 | GI_62642846-S | ILMN_63991 |
| Lyn | 2.06 | GI_13540676-S | ILMN_52908 |
| Malt1_predicted | 1.57 | GI_62664536-S | ILMN_63243 |
| Map3k14_predicted | 0.62 | GI_62657173-S | ILMN_70305 |
| Max | 0.65 | GI_11559987-S | ILMN_53697 |
| Mei1_predicted | 0.66 | GI_62652809-S | ILMN_64977 |
| Mfrp_predicted | 0.55 | GI_34863202-S | ILMN_59378 |
| Mgat5b_predicted | 0.53 | GI_62657438 | ILMN_63750 |
| MGC95065 | 2.11 | GI_57528406-S | ILMN_55559 |
| Mkrn3_predicted | 0.65 | GI_62640437-S | ILMN_48691 |
| Mlze_predicted | 0.49 | GI_62652495-S | ILMN_49201 |
| Mmp11 | 1.58 | GI_6981211-S | ILMN_55582 |
| Mpp4 | 1.62 | GI_10864070-S | ILMN_58699 |
| Msx1 | 0.63 | GI_13592000-S | ILMN_51890 |
| Mte1 | 0.41 | GI_48675861-S | ILMN_63517 |
| Mtmr3_predicted | 0.45 | GI_58865643-A | ILMN_52478 |
| Mtus1 | 1.81 | GI_62662696-S | ILMN_53241 |
| Muted_predicted | 0.63 | GI_62663424-S | ILMN_53780 |
| Myo1e | 1.55 | GI_27465532-S | ILMN_54229 |
| Myrip | 0.65 | GI_33469060-S | ILMN_50156 |
| Naaa | 1.6 | GI_58219536-S | ILMN_52995 |
| Nab1 | 1.54 | GI_12408307-S | ILMN_69831 |
| Ncoa5_predicted | 0.58 | GI_62646372-S | ILMN_58832 |
| Neil1_predicted | 1.52 | GI_62653727-S | ILMN_67504 |
| Nes | 0.66 | GI_6981261-S | ILMN_53585 |
| Neu4_predicted | 2.11 | GI_62655424-S | ILMN_68731 |
| Nkrp2 | 0.56 | GI_19424149-S | ILMN_51410 |
| Nos3 | 0.65 | GI_46409655-S | ILMN_65034 |
| Oas1c | 1.93 | GI_57222311-S | ILMN_56594 |
| Ocm | 2.03 | GI_39930605-S | ILMN_61126 |
| Olr1073_predicted | 1.54 | GI_47578010-S | ILMN_60039 |
| Olr112_predicted | 1.92 | GI_47577934-S | ILMN_67811 |
| Olr1288_predicted | 0.34 | GI_47576286-S | ILMN_52840 |
| Olr1384 | 1.93 | GI_50541942-S | ILMN_49853 |
| Olr1392_predicted | 2.02 | GI_47576118-S | ILMN_63598 |
| Olr1609_predicted | 2.18 | GI_47576482 | ILMN_52502 |
| Olr1726_predicted | 1.5 | GI_47576712-S | ILMN_58633 |
| Olr194_predicted | 2.16 | GI_47576804-S | ILMN_54871 |
| Olr220_predicted | 1.78 | GI_47576874-S | ILMN_61576 |
| Olr247_predicted | 0.59 | GI_47576938-S | ILMN_61039 |
| Olr450_predicted | 2.02 | GI_47577404-S | ILMN_68771 |
| Olr488_predicted | 0.42 | GI_47577498-S | ILMN_70082 |
| Olr790_predicted | 1.74 | GI_47576970-S | ILMN_64238 |
| Olr851_predicted | 0.65 | GI_47576666-S | ILMN_50576 |
| Olr855_predicted | 0.47 | GI_47576640-S | ILMN_49654 |
| Otop1 | 0.23 | GI_31088855-S | ILMN_67544 |
| Pak4_predicted | 0.56 | GI_62639248-S | ILMN_68352 |
| Panx1 | 4.11 | GI_40786482-S | ILMN_61939 |
| Pcca | 0.31 | GI_62662061-S | ILMN_59475 |
| Pcdh16_predicted | 0.62 | GI_62641085-S | ILMN_68871 |
| Pcdha13 | 1.71 | GI_40789246-S | ILMN_50965 |
| Pcdha6 | 0.48 | GI_54019435-S | ILMN_67908 |
| Pcdhb3_predicted | 0.64 | GI_62339316-S | ILMN_55692 |
| Pck1 | 1.57 | GI_61835220-S | ILMN_50651 |
| Pgam2 | 0.66 | GI_8393947-S | ILMN_62722 |
| Phf2_predicted | 1.73 | GI_62663326-S | ILMN_59140 |
| Pi16_predicted | 0.62 | GI_62665752-S | ILMN_64406 |
| Pla2g5 | 0.63 | GI_8393973-S | ILMN_58717 |
| Plac9_predicted | 6.2 | GI_34876899-S | ILMN_52653 |
| Plxna4_predicted | 1.9 | GI_62646990-S | ILMN_68117 |
| Polr3f_predicted | 1.75 | GI_62645963-S | ILMN_61603 |
| Pou6f1 | 1.58 | GI_62653080-S | ILMN_56034 |
| Ppm1h_predicted | 0.64 | GI_62652228 | ILMN_58591 |
| Prkr | 1.49 | GI_9506992-A | ILMN_47830 |
| Prm2 | 1.75 | GI_6981407-S | ILMN_64924 |
| Prph2 | -8.42 | GI_6981467-S | ILMN_57248 |
| Ptger1 | 0.64 | GI_6981431-S | ILMN_67188 |
| Ptger3 | 0.54 | GI_6981433-S | ILMN_69471 |
| Rab6ip2 | 1.59 | GI_31542603-S | ILMN_67212 |
| Rapgef6_predicted | 0.63 | GI_62656083-S | ILMN_70303 |
| Rasgrf2 | 0.63 | GI_16758537-S | ILMN_62709 |
| Rasl10b_predicted | 1.7 | GI_62656675-S | ILMN_62783 |
| Rem1_predicted | 2.32 | GI_62646063-S | ILMN_66497 |
| Rfx3_predicted | 3.11 | GI_62642065-I | ILMN_49420 |
| RGD1304580 | 1.55 | GI_57164138-S | ILMN_56258 |
| RGD1304670_predicted | 0.63 | GI_62641875 | ILMN_62926 |
| RGD1309459 | 0.63 | GI_56119163-S | ILMN_68759 |
| Rgs1 | 0.51 | GI_62659448-S | ILMN_48486 |
| Rhbdl2_predicted | 0.53 | GI_62649599-S | ILMN_60526 |
| Rhced | 1.63 | GI_40254767-S | ILMN_50567 |
| Riok3_predicted | 0.59 | GI_62664046-S | ILMN_53005 |
| Rnf151_predicted | 2.16 | GI_62655835-S | ILMN_57140 |
| Rpl10l_predicted | 1.51 | GI_62650758-S | ILMN_53060 |
| RT1-M1-2 | 2.39 | GI_57012409-S | ILMN_54312 |
| RT1-N2 | 0.64 | GI_62665549-S | ILMN_54668 |
| Rundc1_predicted | 0.41 | GI_62657073-S | ILMN_63936 |
| Runx3 | 1.83 | GI_18426855-S | ILMN_54863 |
| S100a5_predicted | 0.61 | GI_62643825-S | ILMN_67049 |
| Sag | 10.55 | GI_6981499-S | ILMN_58249 |
| Sema4d_predicted | 0.6 | GI_62663283-S | ILMN_56606 |
| Sgp158 | 1.83 | GI_34858291-S | ILMN_60496 |
| Slc22a2 | 1.72 | GI_13994170-S | ILMN_60219 |
| Slc24a5_predicted | 1.52 | GI_62645791-S | ILMN_52959 |
| Slc2a1 | 1.51 | GI_20301951-S | ILMN_56823 |
| Slc2a10_predicted | 2.01 | GI_62646380-S | ILMN_59279 |
| Slc39a12_predicted | 1.6 | GI_62663925-S | ILMN_61465 |
| Slco1a5 | 0.6 | GI_13540641-S | ILMN_66737 |
| Slfn3 | 1.68 | GI_16758505-S | ILMN_50410 |
| Smad5 | 0.63 | GI_11067422-S | ILMN_68848 |
| Smad6_predicted | 1.53 | GI_62653803-S | ILMN_48473 |
| Sostdc1 | 0.56 | GI_24899634-S | ILMN_63695 |
| Sox18_predicted | 1.54 | GI_27705575-S | ILMN_69156 |
| Spata18 | 1.72 | GI_51491895-S | ILMN_66927 |
| Ssa2_predicted | 2.25 | GI_62659440-S | ILMN_48130 |
| Stk35_predicted | 0.56 | GI_62645854-S | ILMN_55921 |
| Strn | 1.69 | GI_62650226-A | ILMN_49273 |
| Stx16_predicted | 1.64 | GI_62646467-S | ILMN_64172 |
| Sult2a1 | 1.86 | GI_62638954-I | ILMN_52264 |
| Syncrip_predicted | 1.55 | GI_62654116-S | ILMN_64105 |
| Syngr4_predicted | 1.55 | GI_62639766-S | ILMN_64786 |
| Synpo | 0.42 | GI_11067428-S | ILMN_59961 |
| Taar8c | 2.24 | GI_28212217-S | ILMN_65208 |
| Tacr3 | 1.52 | GI_8394407-S | ILMN_56816 |
| Tada3l_predicted | 1.59 | GI_34876373-S | ILMN_47956 |
| Tcf12 | 1.7 | GI_6981635-A | ILMN_52980 |
| Tead2 | 0.63 | GI_62639723-S | ILMN_63523 |
| Tgfb1i1 | 1.59 | GI_62641358-S | ILMN_59428 |
| Tm7sf1_predicted | 0.58 | GI_62663701-S | ILMN_63856 |
| Tmc4_predicted | 0.65 | GI_62638819-S | ILMN_68268 |
| Tmem27 | 0.44 | GI_10198601-S | ILMN_55252 |
| Tmod4_predicted | 1.55 | GI_62643878-S | ILMN_69578 |
| Tnni2 | 0.63 | GI_8394465-S | ILMN_58806 |
| Tpd52l2 | 0.61 | GI_38454225-S | ILMN_56259 |
| Trim25_mapped | 1.49 | GI_57164028-S | ILMN_65283 |
| Ttr | 0.53 | GI_6981683-S | ILMN_53794 |
| Twist1 | 0.63 | GI_16758293-S | ILMN_49649 |
| Ucp3 | 1.71 | GI_48675842-S | ILMN_48924 |
| Unc5d_predicted | 0.54 | GI_62662798-S | ILMN_47954 |
| Ush1c | 2 | GI_47058983-S | ILMN_61112 |
| Usp24_predicted | 2.74 | GI_62649323-S | ILMN_68810 |
| Usp29_predicted | 0.53 | GI_62638837 | ILMN_69230 |
| Usp49_predicted | 0.5 | GI_61557195-S | ILMN_69517 |
| Ust_predicted | 0.45 | GI_62638212-S | ILMN_60958 |
| V1re7 | 3.78 | GI_57114273-S | ILMN_52215 |
| Vax1 | 0.26 | GI_12018333-S | ILMN_57171 |
| Vof16 | 0.56 | GI_22219439-S | ILMN_57985 |
| Wisp2 | 0.63 | GI_13928801-S | ILMN_60210 |
| Wnt5a | 1.83 | GI_12018323-S | ILMN_57730 |
| Zfp212_predicted | 0.46 | GI_62647195-S | ILMN_55087 |
| Zfp346_predicted | 0.63 | GI_62663224-S | ILMN_69971 |
| Zfp509_predicted | 1.5 | GI_62660681-S | ILMN_67270 |
| Zfp597 | 1.97 | GI_24308495-S | ILMN_54677 |
| Znf608_predicted | 0.66 | GI_62664428-S | ILMN_50656 |
| Znf629_predicted | 0.64 | GI_62641334-S | ILMN_58285 |
|  | 0.58 | Rn.133548 | ILMN_161336 |
|  | 1.53 | Rn.3659 | ILMN_161556 |
