# Supplementary figures and images for: Effects of Xiaoyaosan on the Hippocampal Gene Expression Profile in Rats Subjected to Chronic Immobilization Stress
Source: Front Psychiatry. 2019 Apr 12;10:178. doi: 10.3389/fpsyt.2019.00178 (PMC6474260; doi:10.3389/fpsyt.2019.00178)

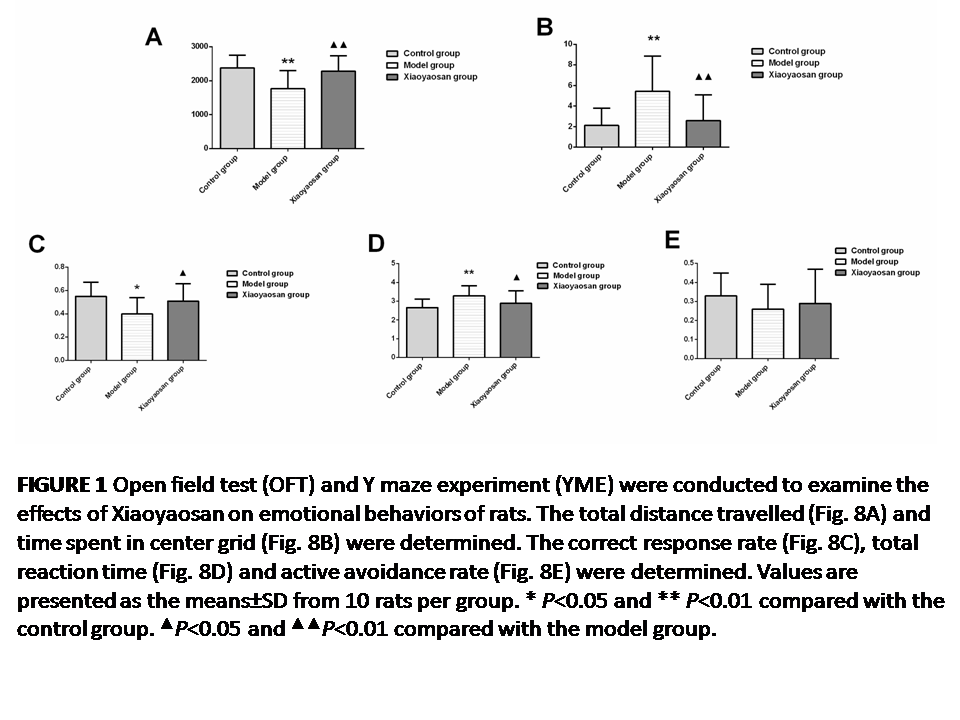

Supplement: Supplementary file 3 [file Image_1.tif]
